# Systemic immune-inflammation index and bone mineral density in postmenopausal women: A cross-sectional study of the national health and nutrition examination survey (NHANES) 2007-2018

**DOI:** 10.3389/fimmu.2022.975400

**Published:** 2022-09-08

**Authors:** Yuchen Tang, Bo Peng, Jinmin Liu, Zhongcheng Liu, Yayi Xia, Bin Geng

**Affiliations:** ^1^ Department of Orthopaedics, Lanzhou University Second Hospital, Lanzhou, Gansu, China; ^2^ Orthopaedics Key Laboratory of Gansu Province, Lanzhou, Gansu, China; ^3^ Orthopaedic Clinical Research Center of Gansu Province, Lanzhou, Gansu, China

**Keywords:** systemic immune-inflammation index, bone mineral density, osteoporosis, osteopenia, postmenopausal women

## Abstract

**Background:**

This study aimed to investigate the association between the systemic immune-inflammation index (SII) and bone mineral density (BMD) and to determine the association between the SII and the risk of osteopenia/osteoporosis among postmenopausal women aged ≥50 years.

**Methods:**

Postmenopausal women aged ≥50 years from the National Health and Nutrition Examination Survey were included. BMD testing was performed using dual-energy X-ray absorptiometry. The SII was calculated based on lymphocyte (LC), neutrophil (NC), and platelet (PC) counts. Moreover, the associations of BMD with SII and other inflammatory markers, including platelet-to-lymphocyte ratio (PLR), neutrophil-to-lymphocyte ratio (NLR), the product of platelet count and neutrophil count (PPN), PC, NC, and LC, were assessed using a multivariable weighted linear regression model. Additionally, the associations of low BMD/osteoporosis with SII and other inflammatory markers were assessed using multivariable weighted logistic regression.

**Results:**

Finally, a total of 893 postmenopausal women with a weighted mean age of 60.90 ± 0.26 years were included finally. This study found that SII was negatively associated with total femur BMD and femoral neck BMD, and postmenopausal women in a higher SII quarter group showed low lumbar spine BMD than the lowest SII quarter group when SII was converted from a continuous variable to a categorical variable. Moreover, increased SII was associated with an increased risk of low BMD and osteoporosis. In addition, this study observed that other inflammatory markers, especially NLR and PPN, were negatively associated with BMD and positively associated with the risk of osteoporosis. Finally, the subgroup analysis showed that the associations between BMD and inflammatory markers were pronounced in postmenopausal women aged ≥65 years or those with normal BMI (<25 kg/m^2^).

**Conclusion:**

SII may be a valuable and convenient inflammatory marker that could be applied to predict the risk of low BMD or osteoporosis among postmenopausal women aged ≥50. Moreover, postmenopausal women with a high level of SII or other inflammatory markers, such as NLR and PPN, should be aware of the potential risk of osteoporosis. However, given the inherent limitations of the present study, additional large-scale studies are required to investigate the role of SII in osteoporosis further.

## Introduction

Osteoporosis, which is characterized by reduced bone mineral density (BMD) and bone microstructure degradation, has become a common public health issue (1). According to previous studies, approximately one-third of females and one-fifth of males aged 50 years and above are at risk of osteoporosis, and the prevalence of osteoporosis is still increasing annually in the middle-aged and elderly population (2–4). Therefore, prevention of osteoporosis has become a major problem faced in modern medicine (1, 5). Osteoporosis is a complex chronic disease characterized by both genetic and environmental factors (1, 6). Moreover, osteoporosis risk assessment has become an essential factor in the prevention of osteoporosis (7, 8). Therefore, finding novel osteoporosis risk factors or biomarkers to evaluate the risk of osteoporosis is receiving increasing attention and is expected to open new preventive avenues.

Several studies have demonstrated that systemic immune and inflammatory status are well associated with osteoporosis (9, 10), which might result from the direct or indirect influence of immune cells on the physiological processes of bone cells (11, 12). For example, rheumatoid arthritis (RA), a common chronic autoimmune disorder, is a risk factor for osteoporosis (13). Glucocorticoids are widely used in clinical practice because of their metabolic and immunosuppressive effects and are associated with significant bone loss (14). Moreover, several previous studies observed that some indices derived from immune cell counts, which reflect the systemic immune and inflammatory status, might be associated with the risk of osteoporosis (15, 16). Öztürk et al. found that an increased neutrophil-lymphocyte ratio was associated with the increased risk of osteoporosis among individuals aged 65 years or older (16). Therefore, the search for a novel index based on immune cell counts to evaluate the risk of osteoporosis may hold great promise for preventing osteoporosis.

The systemic immune-inflammation index (SII) is a novel index based on the lymphocyte, neutrophil, and platelet counts. Accumulating evidence demonstrates that the SII is a useful index to reflect the systemic immune and inflammatory status of the human body (17–20). Moreover, previous studies have found that the SII has potential applications in disease risk and prognosis assessment (17–20), especially in neoplastic diseases. For example, Jomrich et al. found that an increased SII was independently associated with poor prognosis in patients with gastroesophageal adenocarcinomas (19). Hu et al. demonstrated that a high SII score (≥330) was associated with poor prognosis in patients with hepatocellular carcinoma after surgery (18). Additionally, some studies have also observed that an increased SII is a significant risk factor for nonneoplastic diseases. For instance, Qin et al. observed that an increased SII was associated with an increased risk of albuminuria among adults (20). However, owing to the limited number of studies (21), the relationship between BMD and SII remains uncertain, and the role of SII in osteoporosis, especially among postmenopausal females, remains unclear and requires further investigation.

Based on the above-described theoretical background, this study aimed to investigate the association between SII and BMD and to determine the association between SII and the risk of osteopenia/osteoporosis among postmenopausal women aged ≥50 years. We hypothesized that SII was negatively associated with BMD and that the an increased SII would be associated with an increased risk of osteopenia/osteoporosis.

## Materials and methods

### Study population

All subject information was extracted from the National Health and Nutrition Examination Survey (NHANES), which aimed to evaluate the nutrition and health status of general United States (US) residents and was based on a cross-sectional design. The NHANES is affiliated with the Centers for Disease Control and Prevention (USA) and is updated biennially. We extracted data from the NHANES 2007-2018 (2007-2008, 2009-2010, 2013-2014, and 2017-2018. Considering there were no available BMD data in NHANES 2011-2012 and NHANES 2015-2016). The inclusion criteria were as follows: (i) postmenopausal women aged ≥50 years and (ii) participants with complete BMD and SII data. The exclusion criteria were as follows: (i) participants who were pregnant, (ii) participants who were diagnosed with RA by doctors, (iii) participants who were diagnosed with cancer by doctors, (iv) participants who had a history of female hormone use, and (v) participants who had a history of glucocorticoid use. All individuals included in this study provided informed consent, and the ethics review board of the National Center for Health Statistics approved the study (22).

### Menopausal status definitions

Menopausal status was defined based on the self-reported reproductive health questionnaire. Females were regarded as postmenopausal who answered “no” to the question “Have you had at least one menstrual period in the past 12 months?” and subsequently answered “hysterectomy” or “menopause/change of life” to the question “What is the reason that you have not had a period in the past 12 months?”. The details of the self-reported reproductive health questionnaire are available on the NHANES website (23).

### BMD testing and low BMD

All participants (included in the final analysis) underwent BMD testing by dual-energy X-ray absorptiometry (DXA) examinations, which were conducted by certified radiology technologists using Hologic QDR-4500A fan-beam densitometers (Hologic; Bedford, MA, USA). All DXA examination data were analyzed using Hologic APEX software (version 4.0). Other details are provided on the NHANES website (24). Additionally, all participants were divided into three groups (normal, osteopenia, and osteoporosis) according to the total femur (TF), femoral neck (FN), and lumbar spine (LS) BMD. Osteopenia and osteoporosis were defined as previously described by Looker et al. (25, 26). The mean BMD of white females aged 20–29 years was used as the reference value. Individuals with any BMD score of 2.5 standard deviations or more below the norm were considered osteoporosis, individuals with all BMD values of 1.0 standard deviations or more above the norm were considered normal BMD, and other cases were considered osteopenia. Finally, we collectively referred to subjects with osteoporosis or osteopenia as having a low BMD. Details are listed in **Supplementary Table S1**.

### Systemic immune-inflammation index

**Figure 1:**
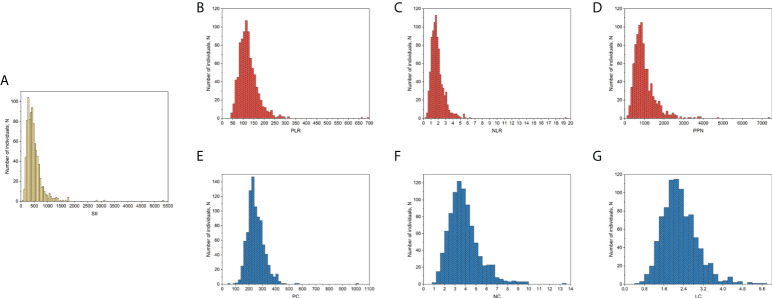
Distribution of SII and other inflammatory markers among postmenopausal women included in the final analysis. **(A)** SII; **(B)** PLR; **(C)**, NLR; **(D)** PPN; **(E)** PC; **(F)**, NC; **(G)**, LC. PC, NC, and LC were measured in 1000 cells/μL. LC, lymphocyte count; NC, neutrophil count; NLR, neutrophil-to-lymphocyte ratio; PC, platelet count; PLR, platelet-to-lymphocyte ratio; PPN, the product of platelet count and neutrophil count; SII, systemic immune-inflammation index.

SII was calculated based on the results of the complete blood count test. The laboratory methodology of the complete blood count test is provided on the NHANES website (27). Moreover, plate count (PC), neutrophil count (NC), and lymphocyte count (LC) were measured in 1000 cells/μL, and the SII was calculated as PC * (NC/LC), according to previous studies (18, 20). For a more comprehensive assessment of the association between SII and BMD, we similarly assessed the association of BMD and other inflammatory markers derived from PC, NC, and LC, including platelet-to-lymphocyte ratio (PLR), neutrophil-to-lymphocyte ratio (NLR), the product of platelet count and neutrophil count (PPN), PC, NC, and LC. In addition, SII, PLR, NLR, PPN, PC, NC, and LC were log2-transformed when conducting regression analysis, considering that these inflammatory markers were right-skewed distributed among postmenopausal women included finally (**Figure 1**).

### Covariates

Considering the potential impact of other factors on bone metabolism, this study also included covariates in the analysis. The selection of covariates available in the NHANES database was based on previous studies (1, 6). Finally, age, race, education level, income level, body mass index (BMI), smoke status, alcohol consumption, diabetes, physical activity level, family history of osteoporosis, milk product consumption, alanine transaminase (ALT), aspartate transaminase (AST), blood calcium, serum creatinine, and serum 25-hydroxyvitamin D [25(OH)D] were considered to be potential covariates in the present study. Detailed information on covariates is provided in **Supplementary Table S2**.

### Statistical analysis

First, all analyses were based on participants with complete data; therefore, individuals with missing covariate data were excluded from the final analysis. Second, the baseline characteristics were indicated by the weighted mean and standard error (SE) (continuous variables) and weighted proportion (categorical variables). The selection of weights used for analysis referenced the instructions provided on the NHANES database (28). Therefore, we used the mobile examination center (MEC) exam weight (WTMEC2YR) for analysis because some of the variables included in the present study were collected in the MEC. Moreover, the sample weight used in the final analysis was equal to one-fourth the value of “WTMEC2YR” because we combined four NHANES survey cycles. Third, the association between the SII and BMD was evaluated using multivariable weighted linear regression models, and the nonlinear relationship between the SII and BMD was characterized by smooth curve fitting and generalized additive models. In addition, the association between SII and bone status (normal vs. low BMD; non-osteoporosis vs. osteoporosis) was assessed using multivariable weighted logistic regression. Fourth, subgroup analysis was conducted with stratified factors, including age (<65; ≥65 years), race (non-Hispanic white, non-Hispanic black, Mexican American, and other races), and BMI (normal, overweight, obese). All analyses were performed using the R software (version 4.0.3; https://www.R-project.org) and EmpowerStats (version 2.0; http://www.empowerstats.com). Statistical significance was set at P < 0.05.

## Results

### Participant selection and baseline characteristics

**Figure 2:**
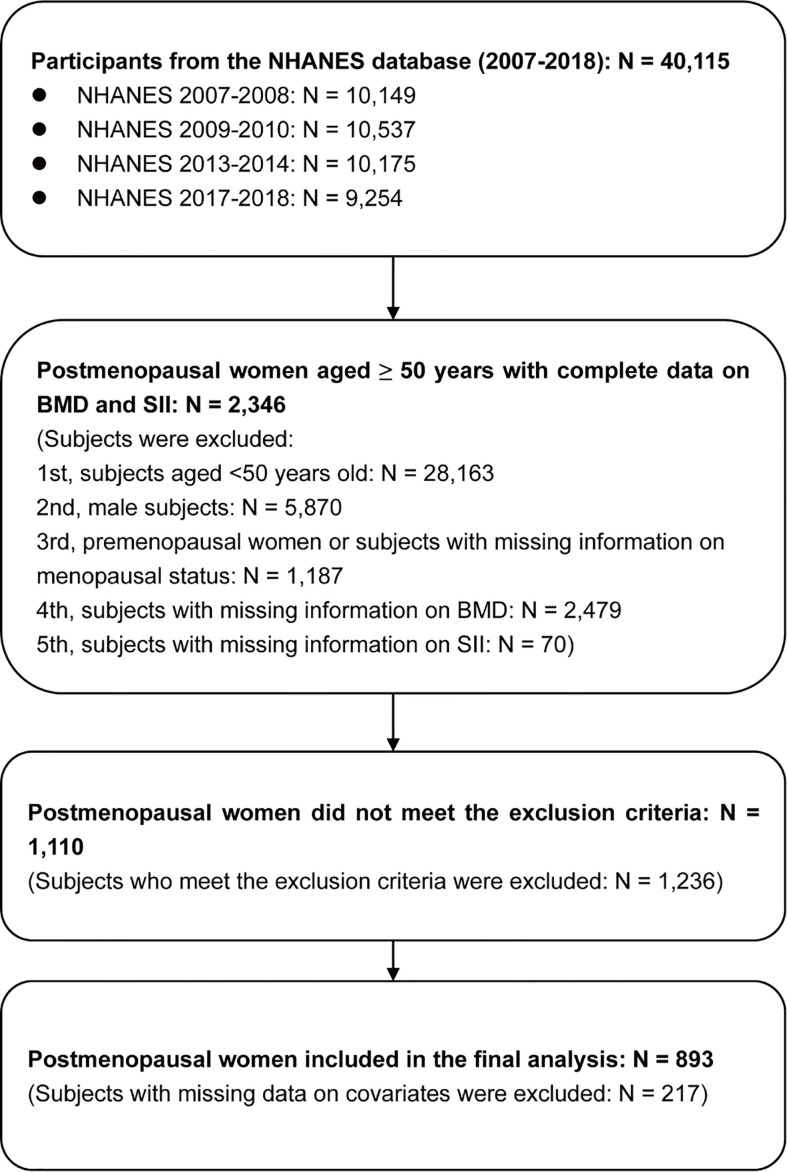
Flowchart of participant selection. BMD, bone mineral density; NHANES, National Health and Nutrition Examination Survey; SII, systemic immune-inflammation index.

The flowchart of participant selection is shown in **Figure 2**. The information of 40,115 participants was extracted from the NHANES (2007-2008: N=10,149; 2009-2010: N=10,537; 2013-2014: N=10,175; 2017-2018: N=9,254). First, we excluded subjects aged <50 years (N=28,163) and male participants (N=5,870). Moreover, premenopausal females and subjects with missing information on menopausal status (N=1,187) were excluded from the present study. Additionally, we excluded postmenopausal females with missing information on BMD (N=2,479) and SII (N=70). Second, participants who met the exclusion criteria (N=1,236) were excluded. Third, participants with missing covariate information (n=217) were excluded. Finally, 893 postmenopausal females aged ≥50 years were included in the final analysis, and weighted samples of postmenopausal females aged ≥50 years represent a population of 7,854,530.

**Figure 3:**
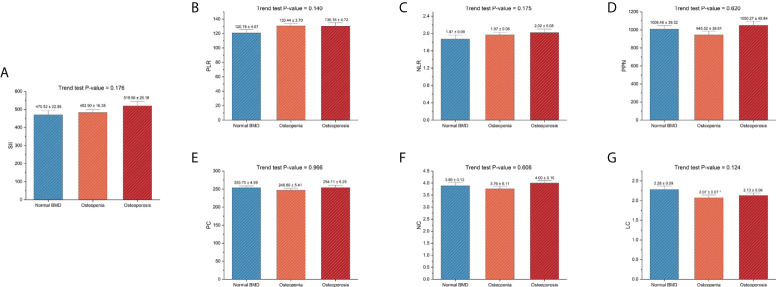
Differences in SII and other inflammatory markers among different skeletal status groups. **(A)** SII; **(B)** PLR; **(C)**, NLR; **(D)** PPN; **(E)** PC; **(F)**, NC; **(G)**, LC. PC, NC, and LC were measured in 1000 cells/μL. BMD, bone mineral density; LC, lymphocyte count; NC, neutrophil count; NLR, neutrophil-to-lymphocyte ratio; PC, platelet count; PLR, platelet-to-lymphocyte ratio; PPN, the product of platelet count and neutrophil count; SII, systemic immune-inflammation index. *P-value < 0.05 compared with the normal BMD group.

The baseline characteristics are listed in **Table 1**. All postmenopausal women included in the final analysis had a weighted mean age of 60.90 ± 0.26 years. The mean TF-BMD, FN-BMD, and LS-BMD were 0.85 ± 0.01, 0.71 ± 0.01, 0.93 ± 0.01, respectively. Moreover, we compared SII and other inflammatory markers among postmenopausal women with normal BMD, osteopenia, and osteoporosis. The results showed that postmenopausal women with osteopenia showed a higher level of LC than those with normal BMD. Moreover, there were no significant differences in other inflammatory markers between women with normal BMD and those with osteopenia/osteoporosis. The results are listed in **Figure 3**.

**Table 1:** Baseline characteristics of postmenopausal women included in the final analysis.

Characteristics		Mean or proportion
Age [year], mean (SE)		60.90 (0.26)
Race, n (%)	Non-Hispanic white	323 (66.96)
	Mexican American	153 (6.61)
	Other Hispanic	106 (5.32)
	Non-Hispanic black	191 (12.21)
	Other races	120 (8.90)
Education level, n (%)	Under high school	264 (17.85)
	High school or equivalent	221 (29.92)
	Above high school	408 (52.23)
Income level [PIR], mean (SE)		3.02 (0.08)
BMI, n (%)	Normal	262 (33.07)
	(BMI <25 kg/m2)	
	Overweight	308 (32.91)
	(25≤ BMI < 30 kg/m2)	
	Obese	323 (34.02)
	(BMI ≥ 30 kg/m2)	
Smoke status, n (%)	Current smokers	125 (14.16)
	Quit smoking	179 (22.04)
	Never smoke	589 (63.80)
Alcohol consumption, n (%)	Yes	404 (52.81)
	(≥ once monthly)	
	No	489 (47.19)
	(< once monthly)	
Milk product consumption, n (%)	Never	198 (23.55)
	Rarely (less than once a week)	144 (13.63)
	Sometimes (once a week or more, but less than once a day)	217 (22.07)
	Often (once a day or more)	330 (40.49)
	Varied	4 (0.26)
Physical activity level, n (%)	NMVPA	316 (29.74)
	(0 MET-mins/week)	
	LMVPA	149 (17.32)
	(1-599 MET-mins/week)	
	MMVPA	111 (15.35)
	(600-1199 MET-mins/week)	
	HMVPA	317 (37.59)
	(≥1200 MET-mins/week)	
Diabetes, n (%)	Yes	163 (12.05)
	No	704 (84.85)
	Borderline	26 (3.10)
Family history of osteoporosis, n (%)	Yes	143 (21.24)
	No	750 (78.76)
ALT [U/L], mean (SE)		22.61 (0.55)
AST [U/L], mean (SE)		23.98 (0.36)
Blood calcium [mg/dL], mean (SE)		9.46 (0.02)
Serum creatinine [mg/dL], mean (SE)		0.81 (0.01)
Serum 25(OH)D [nmol/L], mean (SE)		74.19 (1.32)
TF-BMD [g/cm^2^], mean (SE)		0.85 (0.01)
FN-BMD [g/cm^2^], mean (SE)		0.71 (0.01)
LS-BMD [g/cm^2^], mean (SE)		0.93 (0.01)

%, weighted proportion.

25(OH)D, 25-hydroxyvitamin D, ALT, alanine transaminase; AST, aspartate transaminase; BMD, bone mineral density; BMI, body mass index; FN, femoral neck; HMVPA, high moderate-to-vigorous physical activity; LMVPA, low moderate-to-vigorous physical activity; LS, lumbar spine; MMVPA, medium moderate-to-vigorous physical activity; NMVPA, no moderate-to-vigorous physical activity; PIR, family income-to-poverty ratio; SE, standard error, TF, total femur.

### Association between SII and BMD

**Figure 4:**
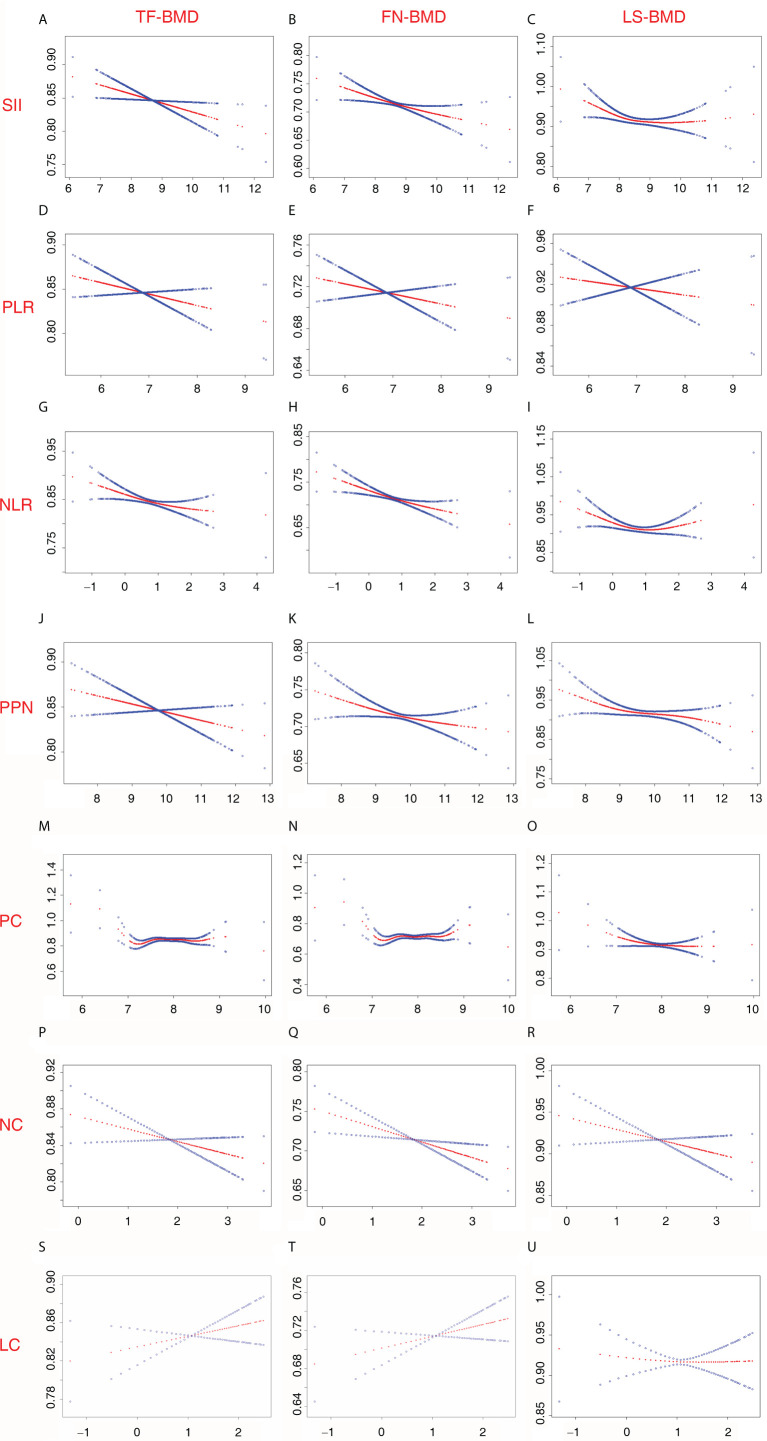
Non-linear relationship between inflammatory marker and BMD among postmenopausal women. **(A)** SII and TF-BMD; **(B)** SII and FN-BMD; **(C)** SII and LS-BMD; **(D)** PLR and TF-BMD; **(E)** PLR and FN-BMD; **(F)** PLR and LS-BMD; **(G)** NLR and TF-BMD; **(H)** NLR and FN-BMD; **(I)** NLR and LS-BMD; **(J)** PPN and TF-BMD; **(K)** PPN and FN-BMD; **(L)** PPN and LS-BMD; **(M)** PC and TF-BMD; **(N)** PC and FN-BMD; **(O)** PC and LS-BMD; **(P)** NC and TF-BMD; **(Q)** NC and FN-BMD; **(R)** NC and LS-BMD; **(S)** LC and TF-BMD; **(T)** LC and FN-BMD; **(U)** LC and LS-BMD. SII, PLR, NLR, PPN, PC, NC, and LCwere considered continuous variables (log2-SII, log2-PLR, log2-NLR, log2-PPN, log2-PC, log2-NC, log2-LC). Age (50-64; 65 and over), race (non-Hispanic white; Mexican American; other Hispanic; non-Hispanic black; other races), education level (under high school; high school or equivalent; above high school), income level (Q1-Q4), BMI (normal; overweight; obese), smoke status (current smokers; quit smoking; never smoke), alcohol consumption (≥ once monthly; < once monthly), diabetes (yes; no; borderline), physical activity level (NMVPA; LMVPA; MMVPA; HMVPA), family history of osteoporosis (yes; no), milk product consumption (never; rarely; sometimes; often; varied), ALT (Q1-Q4); AST (Q1-Q4), blood calcium (Q1-Q4), serum creatinine (Q1-Q4), and serum 25(OH)D (Q1-Q4) were adjusted. 25(OH)D, 25-hydroxyvitamin D; ALT, alanine transaminase; AST, aspartate transaminase; BMD, bone mineral density; BMI, body mass index; FN, femoral neck; HMVPA, high moderate-to-vigorous physical activity; LC, lymphocyte count; LMVPA, low moderate-to-vigorous physical activity; LS, lumbar spine; MMVPA, medium moderate-to-vigorous physical activity; NC, neutrophil count; NLR, neutrophil-to-lymphocyte ratio; NMVPA, no moderate-to-vigorous physical activity; PC, platelet count; PLR, platelet-to-lymphocyte ratio; PPN, the product of platelet count and neutrophil count; SII, systemic immune-inflammation index; TF, total femur.

The associations of BMD with SII and other inflammatory markers are listed in **Table 2**. When no covariates were adjusted (Model 1), log2-NLR was negatively associated with FN-BMD, and no significant association was observed between BMD and other inflammatory markers. Moreover, when age, race, and BMI were adjusted (Model 2), log2-SII, log2-NLR, log2-PPN, and log2-NC were negatively associated with FN-BMD, and log2-PPN and log2-PC were negatively associated with LS-BMD. In addition, when all covariates were adjusted (Model 3), log2-SII and log2-PPN were negatively associated with TF-BMD; log2-SII, log2-NLR, log2-PPN, and log2-NC were negatively associated with FN-BMD; and log2-PPN and log2-PC were negatively associated with LS-BMD. In the sensitivity analysis, SII and other inflammatory markers were converted from a continuous variable to a categorical variable (Q1-Q4). The results from the sensitivity analysis were consistent with the main analysis. Particularly, there was no significant association between SII and LS-BMD when SII was continuous. However, the sensitivity analysis results suggested that postmenopausal women in a higher SII quarter group (second quartile and third quartile) showed low LS-BMD than the lowest SII quarter group. Other details of the sensitivity analysis are listed in **Table 2**. In addition, we assessed the non-linear relationships of BMD with SII and other inflammatory markers derived from PC, NC, and LC. Particularly, we observed that LS-BMD exhibited an inverted U-shaped relationship with log2-SII and los2-NLR. The specific results are shown in **Figure 4**.

**Table 2:** Association of BMD with SII and inflammatory markers.

Index	Outcome	Continuous or categories	Model 1 *				Model 2 ‡				Model 3 ¶			
			β	95%CI low	95%CI upp	P-value	β	95%CI low	95%CI upp	P-value	β	95%CI low	95%CI upp	P-value
SII	TF-BMD	Log2-SII	-0.012	-0.033	0.010	0.285	-0.020	-0.039	-0.000	0.051	**-0.020**	**-0.036**	**-0.004**	**0.025**
		Q1	Reference				Reference				Reference			
		Q2	0.000	-0.031	0.032	0.996	-0.009	-0.039	0.022	0.584	-0.008	-0.037	0.022	0.607
		Q3	-0.028	-0.069	0.013	0.183	-0.033	-0.069	0.002	0.066	-0.031	-0.064	0.002	0.078
		Q4	-0.018	-0.053	0.017	0.318	-0.030	-0.064	0.003	0.083	-0.030	-0.059	-0.000	0.065
	FN-BMD	Log2-SII	-0.002	-0.026	0.023	0.889	**-0.018**	**-0.034**	**-0.003**	**0.024**	**-0.020**	**-0.033**	**-0.006**	**0.011**
		Q1	Reference				Reference				Reference			
		Q2	-0.011	-0.044	0.021	0.498	-0.014	-0.046	0.018	0.384	-0.014	-0.045	0.017	0.381
		Q3	-0.032	-0.067	0.003	0.078	**-0.031**	**-0.059**	**-0.002**	**0.041**	**-0.031**	**-0.059**	**-0.003**	**0.040**
		Q4	-0.025	-0.053	0.003	0.084	**-0.028**	**-0.056**	**-0.001**	**0.048**	**-0.029**	**-0.055**	**-0.004**	**0.036**
	LS-BMD	Log2-SII	-0.002	-0.026	0.023	0.889	-0.012	-0.036	0.012	0.328	-0.017	-0.039	0.006	0.166
		Q1	Reference				Reference				Reference			
		Q2	-0.033	-0.068	0.002	0.066	**-0.042**	**-0.079**	**-0.004**	**0.034**	**-0.045**	**-0.082**	**-0.007**	**0.029**
		Q3	-0.031	-0.069	0.008	0.122	**-0.040**	**-0.078**	**-0.003**	**0.038**	**-0.040**	**-0.073**	**-0.007**	**0.028**
		Q4	-0.006	-0.048	0.036	0.783	-0.023	-0.063	0.016	0.253	-0.033	-0.073	0.007	0.127
PLR	TF-BMD	Log2-PLR	-0.023	-0.055	0.010	0.171	-0.013	-0.040	0.015	0.369	-0.018	-0.040	0.003	0.114
		Q1	Reference				Reference				Reference			
		Q2	0.013	-0.030	0.057	0.545	0.005	-0.031	0.041	0.776	0.005	-0.025	0.035	0.736
		Q3	-0.007	-0.043	0.029	0.691	-0.005	-0.037	0.026	0.745	-0.008	-0.034	0.017	0.529
		Q4	-0.025	-0.065	0.014	0.216	-0.015	-0.050	0.019	0.392	-0.023	-0.051	0.005	0.125
	FN-BMD	Log2-PLR	-0.017	-0.044	0.009	0.194	-0.007	-0.029	0.015	0.521	-0.012	-0.031	0.007	0.216
		Q1	Reference				Reference				Reference			
		Q2	0.014	-0.024	0.053	0.472	0.011	-0.021	0.043	0.517	0.007	-0.021	0.035	0.648
		Q3	-0.011	-0.045	0.024	0.549	-0.006	-0.035	0.023	0.696	-0.011	-0.035	0.013	0.389
		Q4	-0.014	-0.048	0.020	0.429	-0.002	-0.031	0.027	0.897	-0.011	-0.036	0.014	0.405
	LS-BMD	Log2-PLR	-0.002	-0.035	0.032	0.926	0.002	-0.029	0.033	0.909	-0.005	-0.031	0.022	0.725
		Q1	Reference				Reference				Reference			
		Q2	-0.011	-0.058	0.037	0.659	-0.019	-0.066	0.028	0.435	-0.024	-0.064	0.016	0.255
		Q3	-0.011	-0.054	0.032	0.613	-0.011	-0.050	0.027	0.569	-0.014	-0.048	0.019	0.410
		Q4	-0.006	-0.050	0.038	0.779	-0.005	-0.045	0.036	0.818	-0.016	-0.051	0.018	0.367
NLR	TF-BMD	Log2-NLR	-0.017	-0.039	0.006	0.151	-0.016	-0.036	0.005	0.143	-0.017	-0.034	0.001	0.077
		Q1	Reference				Reference				Reference			
		Q2	**-0.038**	**-0.070**	**-0.007**	**0.020**	**-0.039**	**-0.067**	**-0.011**	**0.008**	**-0.036**	**-0.065**	**-0.007**	**0.023**
		Q3	**-0.041**	**-0.074**	**-0.009**	**0.015**	**-0.037**	**-0.067**	**-0.007**	**0.018**	**-0.035**	**-0.063**	**-0.008**	**0.021**
		Q4	-0.029	-0.068	0.010	0.149	-0.031	-0.067	0.004	0.091	-0.031	-0.062	0.000	0.067
	FN-BMD	Log2-NLR	**-0.024**	**-0.042**	**-0.006**	**0.012**	**-0.017**	**-0.034**	**-0.001**	**0.041**	**-0.018**	**-0.033**	**-0.004**	**0.024**
		Q1	Reference				Reference				Reference			
		Q2	**-0.045**	**-0.075**	**-0.014**	**0.005**	**-0.040**	**-0.066**	**-0.014**	**0.005**	**-0.035**	**-0.061**	**-0.008**	**0.019**
		Q3	**-0.041**	**-0.073**	**-0.009**	**0.015**	**-0.032**	**-0.061**	**-0.002**	**0.040**	-0.030	-0.059	-0.002	0.050
		Q4	**-0.042**	**-0.074**	**-0.010**	**0.013**	**-0.036**	**-0.065**	**-0.006**	**0.021**	**-0.034**	**-0.060**	**-0.008**	**0.020**
	LS-BMD	Log2-NLR	0.003	-0.027	0.033	0.846	-0.000	-0.030	0.030	0.994	-0.005	-0.032	0.021	0.693
		Q1	Reference				Reference				Reference			
		Q2	**-0.041**	**-0.081**	**-0.002**	**0.045**	**-0.045**	**-0.085**	**-0.005**	**0.032**	**-0.039**	**-0.076**	**-0.003**	**0.049**
		Q3	-0.036	-0.072	0.001	0.058	-0.035	-0.071	0.000	0.055	-0.035	-0.067	-0.002	0.051
		Q4	-0.006	-0.058	0.047	0.835	-0.014	-0.064	0.036	0.586	-0.018	-0.062	0.027	0.446
PPN	TF-BMD	Log2-PPN	0.002	-0.019	0.023	0.847	-0.020	-0.039	-0.000	0.051	**-0.018**	**-0.035**	**-0.001**	**0.048**
		Q1	Reference				Reference				Reference			
		Q2	0.003	-0.034	0.039	0.885	-0.008	-0.041	0.024	0.607	-0.018	-0.048	0.012	0.251
		Q3	0.022	-0.006	0.051	0.133	-0.009	-0.041	0.023	0.590	-0.008	-0.039	0.023	0.614
		Q4	0.012	-0.024	0.048	0.523	-0.032	-0.066	0.002	0.073	-0.028	-0.056	-0.000	0.062
	FN-BMD	Log2-PPN	-0.003	-0.020	0.014	0.744	**-0.019**	**-0.035**	**-0.003**	**0.026**	**-0.019**	**-0.033**	**-0.005**	**0.017**
		Q1	Reference				Reference				Reference			
		Q2	-0.005	-0.038	0.029	0.788	-0.011	-0.041	0.020	0.500	-0.022	-0.050	0.007	0.149
		Q3	0.011	-0.014	0.035	0.395	-0.011	-0.038	0.015	0.402	-0.017	-0.043	0.009	0.222
		Q4	-0.003	-0.030	0.024	0.839	**-0.035**	**-0.062**	**-0.009**	**0.012**	**-0.036**	**-0.059**	**-0.013**	**0.006**
	LS-BMD	Log2-PPN	-0.005	-0.029	0.019	0.689	**-0.025**	**-0.048**	**-0.001**	**0.044**	**-0.027**	**-0.049**	**-0.005**	**0.024**
		Q1	Reference				Reference				Reference			
		Q2	-0.010	-0.057	0.037	0.674	-0.022	-0.068	0.024	0.354	-0.027	-0.068	0.015	0.219
		Q3	0.005	-0.035	0.045	0.806	-0.023	-0.067	0.021	0.304	-0.025	-0.067	0.016	0.242
		Q4	0.004	-0.040	0.048	0.863	-0.035	-0.079	0.008	0.120	-0.040	-0.078	-0.002	0.054
PC	TF-BMD	Log2-PC	0.005	-0.042	0.052	0.838	-0.028	-0.068	0.012	0.171	-0.026	-0.059	0.006	0.128
		Q1	Reference				Reference				Reference			
		Q2	0.003	-0.035	0.041	0.890	-0.008	-0.041	0.025	0.633	-0.007	-0.036	0.022	0.640
		Q3	0.026	-0.013	0.064	0.204	-0.017	-0.053	0.018	0.346	-0.022	-0.054	0.010	0.200
		Q4	0.004	-0.040	0.048	0.864	-0.022	-0.062	0.017	0.269	-0.022	-0.054	0.011	0.206
	FN-BMD	Log2-PC	0.011	-0.029	0.052	0.589	-0.019	-0.054	0.016	0.299	-0.020	-0.051	0.011	0.217
		Q1	Reference				Reference				Reference			
		Q2	0.001	-0.035	0.037	0.976	-0.009	-0.041	0.023	0.596	-0.009	-0.036	0.019	0.557
		Q3	0.017	-0.016	0.050	0.310	-0.018	-0.049	0.013	0.255	-0.022	-0.050	0.007	0.150
		Q4	0.011	-0.028	0.051	0.583	-0.013	-0.050	0.024	0.497	-0.016	-0.049	0.018	0.367
	LS-BMD	Log2-PC	-0.015	-0.058	0.028	0.495	**-0.042**	**-0.082**	**-0.003**	**0.041**	**-0.044**	**-0.076**	**-0.012**	**0.013**
		Q1	Reference				Reference				Reference			
		Q2	-0.028	-0.068	0.013	0.189	-0.033	-0.075	0.010	0.137	-0.037	-0.078	0.005	0.100
		Q3	-0.013	-0.052	0.027	0.539	**-0.045**	**-0.086**	**-0.004**	**0.036**	**-0.050**	**-0.092**	**-0.008**	**0.031**
		Q4	-0.012	-0.055	0.030	0.576	-0.034	-0.075	0.006	0.106	**-0.037**	**-0.072**	**-0.002**	**0.050**
NC	TF-BMD	Log2-NC	0.001	-0.023	0.026	0.916	-0.022	-0.047	0.002	0.079	-0.019	-0.040	0.003	0.101
		Q1	Reference				Reference				Reference			
		Q2	-0.006	-0.037	0.025	0.704	-0.011	-0.038	0.015	0.405	-0.015	-0.040	0.009	0.233
		Q3	-0.012	-0.046	0.023	0.509	-0.018	-0.050	0.015	0.292	-0.012	-0.042	0.019	0.464
		Q4	0.003	-0.029	0.035	0.853	-0.036	-0.070	-0.001	0.051	-0.032	-0.062	-0.001	0.054
	FN-BMD	Log2-NC	-0.011	-0.030	0.009	0.295	**-0.026**	**-0.046**	**-0.005**	**0.016**	**-0.024**	**-0.042**	**-0.007**	**0.013**
		Q1	Reference				Reference				Reference			
		Q2	-0.014	-0.045	0.018	0.392	-0.015	-0.045	0.014	0.305	-0.019	-0.045	0.006	0.158
		Q3	-0.022	-0.056	0.012	0.202	-0.022	-0.053	0.009	0.169	-0.017	-0.045	0.012	0.265
		Q4	-0.014	-0.041	0.013	0.300	**-0.041**	**-0.071**	**-0.011**	**0.010**	**-0.040**	**-0.065**	**-0.014**	**0.006**
	LS-BMD	Log2-NC	-0.002	-0.032	0.029	0.921	-0.024	-0.056	0.008	0.142	-0.026	-0.057	0.004	0.101
		Q1	Reference				Reference				Reference			
		Q2	-0.012	-0.046	0.023	0.509	-0.015	-0.049	0.018	0.378	-0.016	-0.046	0.015	0.325
		Q3	-0.009	-0.052	0.034	0.689	-0.017	-0.058	0.024	0.418	-0.016	-0.055	0.023	0.436
		Q4	0.006	-0.041	0.053	0.803	-0.027	-0.075	0.020	0.263	-0.029	-0.074	0.016	0.217
LC	TF-BMD	Log2-LC	0.028	-0.004	0.059	0.091	-0.001	-0.030	0.027	0.919	0.006	-0.016	0.027	0.609
		Q1	Reference				Reference				Reference			
		Q2	0.006	-0.029	0.041	0.728	-0.000	-0.030	0.030	0.994	0.005	-0.025	0.035	0.738
		Q3	0.028	-0.009	0.064	0.147	0.001	-0.034	0.035	0.969	0.006	-0.026	0.037	0.730
		Q4	0.018	-0.025	0.061	0.417	-0.014	-0.053	0.024	0.459	-0.005	-0.036	0.027	0.778
	FN-BMD	Log2-LC	0.025	0.000	0.050	0.053	-0.002	-0.024	0.020	0.830	0.002	-0.015	0.020	0.800
		Q1	Reference				Reference				Reference			
		Q2	-0.006	-0.040	0.029	0.750	-0.013	-0.044	0.019	0.436	-0.011	-0.042	0.021	0.517
		Q3	0.030	-0.006	0.066	0.108	0.004	-0.030	0.039	0.799	0.006	-0.026	0.038	0.717
		Q4	0.016	-0.021	0.053	0.397	-0.015	-0.048	0.017	0.358	-0.009	-0.037	0.018	0.508
	LS-BMD	Log2-LC	-0.006	-0.048	0.036	0.767	-0.026	-0.066	0.014	0.204	-0.020	-0.052	0.012	0.231
		Q1	Reference				Reference				Reference			
		Q2	-0.002	-0.041	0.036	0.911	-0.001	-0.038	0.035	0.943	0.002	-0.029	0.032	0.913
		Q3	-0.020	-0.066	0.025	0.380	-0.037	-0.082	0.007	0.108	-0.031	-0.071	0.008	0.137
		Q4	-0.004	-0.059	0.051	0.891	-0.024	-0.074	0.026	0.346	-0.014	-0.053	0.024	0.481

SII: Q1 (68.67-289.81), Q2 (290.00-419.38), Q3 (419.76-570.94), Q4 (571.17-5313.00); PLR: Q1 (42.65-93.87), Q2 (94.07-115.77), Q3 (115.79-144.29), Q4 (144.38-690.00); NLR: Q1 (0.333-1.278), Q2 (1.281-1.706), Q3 (1.708-2.241), Q4 (2.250-19.250); PPN: Q1 (151.20-638.60), Q2 (639.60-856.80), Q3 (857.50-1173.00), Q4 (1177.40-7352.40); PC: Q1 (54.00-208.00), Q2 (209.00-241.00), Q3 (242.00-285.00), Q4 (286.00-1000.00); NC: Q1 (0.90-2.70), Q2 (2.80-3.50), Q3 (3.60-4.50), Q4 (4.60-13.20); LC: Q1 (0.40-1.60), Q2 (1.70-2.00), Q3 (2.10-2.50), Q4 (2.60-5.60).

Bold fonts indicate P value < 0.05.

Income level, ALT, AST, blood calcium, serum creatinine, and serum 25(OH)D were categorized into four groups according to the quartiles (Q1-Q4) of distribution.

* Model 1: Unadjusted model.

‡ Model 2: Age (50-64; 65 and over), race (non-Hispanic white; Mexican American; other Hispanic; non-Hispanic black; other races), and BMI (normal; overweight; obese) were adjusted.

¶ Model 3: Age (50-64; 65 and over), race (non-Hispanic white; Mexican American; other Hispanic; non-Hispanic black; other races), education level (under high school; high school or equivalent; above high school), income level (Q1-Q4), BMI (normal; overweight; obese), smoke status (current smokers; quit smoking; never smoke), alcohol consumption (≥ once monthly; < once monthly), diabetes (yes; no; borderline), physical activity level (NMVPA; LMVPA; MMVPA; HMVPA), family history of osteoporosis (yes; no), milk product consumption (never; rarely; sometimes; often; varied), ALT (Q1-Q4); AST (Q1-Q4), blood calcium (Q1-Q4), serum creatinine (Q1-Q4), and serum 25(OH)D (Q1-Q4) were adjusted.

25(OH)D, 25-hydroxyvitamin D; ALT, alanine transaminase; AST, aspartate transaminase; BMD, bone mineral density; BMI, body mass index; CI, confidence interval; FN, femoral neck; HMVPA, high moderate-to-vigorous physical activity; LC, lymphocyte count; LMVPA, low moderate-to-vigorous physical activity; LS, lumbar spine; MMVPA, medium moderate-to-vigorous physical activity; NC, neutrophil count; NLR, neutrophil-to-lymphocyte ratio; NMVPA, no moderate-to-vigorous physical activity; PC, platelet count; PLR, platelet-to-lymphocyte ratio; PPN, the product of platelet count and neutrophil count; SII, systemic immune-inflammation index; TF, total femur.

### Association between SII and risk of low BMD/osteoporosis

The associations of the risk of low BMD/osteoporosis with SII and other inflammatory markers are listed in **Table 3**. When no covariates were adjusted (Model 1), increased log2-LC was associated with a decreased risk of low BMD (normal BMD vs. low BMD), while increased log2-NC was associated with an increased risk of osteoporosis (non-osteoporosis vs. osteoporosis). Moreover, when age, race, and BMI were adjusted (Model 2), increased log2-SII, log2-PPN, or log2-NC was associated with an increased risk of osteoporosis. In addition, when all covariates were adjusted (Model 3), increased log2-SII or log2-PLR was associated with an increased risk of low BMD; and increased log2-SII, log2-PPN, or log2-NC was associated with an increased risk of osteoporosis. In the sensitivity analysis, SII and other inflammatory markers derived from PC, NC, and LC were converted from a continuous variable to a categorical variable (Q1-Q4). The results from the sensitivity analysis were consistent with the primary analysis. In particular, there was no significant association between NLR and the risk of osteoporosis when NLR was continuous. However, the sensitivity analysis results suggested that postmenopausal women in a higher NLR quarter group (second quartile and fourth quartile) showed a higher risk of osteoporosis than the lowest NLR quarter group. Other details of the sensitivity analysis are listed in **Table 3**.

**Table 3:** Association of the risk of low BMD/osteoporosis with SII and other inflammatory markers.

Index	Outcome	Continuous or categories	Model 1 *				Model 2 ‡				Model 3 ¶			
			OR	95%CI low	95%CI upp	P-value	OR	95%CI low	95%CI upp	P-value	OR	95%CI low	95%CI upp	P-value
SII	Normal BMD vs. Low BMD	Log2-SII	1.183	0.878	1.595	0.274	1.385	0.974	1.968	0.075	**1.576**	**1.062**	**2.341**	**0.035**
		Q1	Reference				Reference				Reference			
		Q2	0.927	0.509	1.691	0.806	1.001	0.493	2.031	0.998	0.989	0.491	1.993	0.977
		Q3	1.421	0.712	2.836	0.323	1.631	0.778	3.420	0.201	1.803	0.897	3.624	0.114
		Q4	1.093	0.572	2.088	0.788	1.303	0.633	2.684	0.476	1.512	0.686	3.333	0.318
	Non-Osteoporosis vs. Osteoporosis	Log2-SII	1.286	0.980	1.687	0.074	**1.398**	**1.015**	**1.925**	**0.045**	**1.498**	**1.087**	**2.065**	**0.022**
		Q1	Reference				Reference				Reference			
		Q2	**2.124**	**1.133**	**3.982**	**0.022**	**2.350**	**1.057**	**5.222**	**0.041**	**2.673**	**1.150**	**6.213**	**0.034**
		Q3	1.284	0.715	2.306	0.405	1.323	0.690	2.536	0.403	1.298	0.635	2.656	0.483
		Q4	**2.198**	**1.333**	**3.623**	**0.003**	**2.549**	**1.377**	**4.718**	**0.004**	**2.986**	**1.533**	**5.815**	**0.005**
PLR	Normal BMD vs. Low BMD	Log2-PLR	1.479	0.926	2.362	0.107	1.429	0.853	2.394	0.180	**1.739**	**1.077**	**2.808**	**0.034**
		Q1	Reference				Reference				Reference			
		Q2	1.062	0.573	1.967	0.849	1.188	0.557	2.533	0.657	1.406	0.676	2.924	0.373
		Q3	1.171	0.658	2.082	0.593	1.194	0.606	2.356	0.610	1.404	0.761	2.592	0.292
		Q4	1.656	0.883	3.106	0.122	1.625	0.806	3.278	0.181	**2.211**	**1.148**	**4.259**	**0.028**
	Non-Osteoporosis vs. Osteoporosis	Log2-PLR	1.092	0.717	1.664	0.683	0.982	0.603	1.601	0.944	1.199	0.756	1.903	0.449
		Q1	Reference				Reference				Reference			
		Q2	1.046	0.495	2.211	0.906	1.094	0.478	2.503	0.832	1.465	0.616	3.485	0.398
		Q3	1.222	0.611	2.446	0.573	1.147	0.546	2.407	0.719	1.376	0.660	2.870	0.405
		Q4	0.935	0.459	1.904	0.854	0.811	0.365	1.802	0.609	1.140	0.535	2.431	0.738
NLR	Normal BMD vs. Low BMD	Log2-NLR	1.333	0.901	1.972	0.156	1.364	0.885	2.103	0.165	1.493	0.957	2.330	0.092
		Q1	Reference				Reference				Reference			
		Q2	1.738	0.966	3.129	0.070	1.939	1.011	3.721	0.052	1.677	0.877	3.205	0.134
		Q3	1.708	0.872	3.347	0.124	1.798	0.852	3.794	0.130	1.913	0.851	4.300	0.133
		Q4	1.439	0.735	2.818	0.293	1.546	0.732	3.267	0.259	1.594	0.755	3.364	0.236
	Non-Osteoporosis vs. Osteoporosis	Log2-NLR	1.299	0.976	1.729	0.077	1.261	0.880	1.807	0.212	1.352	0.946	1.933	0.113
		Q1	Reference				Reference				Reference			
		Q2	**2.606**	**1.468**	**4.626**	**0.002**	**2.886**	**1.484**	**5.612**	**0.003**	**3.129**	**1.584**	**6.179**	**0.004**
		Q3	**2.127**	**1.334**	**3.392**	**0.002**	**1.979**	**1.175**	**3.333**	**0.013**	1.764	1.008	3.085	0.061
		Q4	**2.178**	**1.206**	**3.934**	**0.012**	**2.193**	**1.090**	**4.414**	**0.032**	**2.610**	**1.357**	**5.020**	**0.010**
PPN	Normal BMD vs. Low BMD	Log2-PPN	0.909	0.687	1.203	0.508	1.215	0.856	1.723	0.281	1.341	0.888	2.025	0.177
		Q1	Reference				Reference				Reference			
		Q2	0.742	0.412	1.336	0.325	0.800	0.391	1.637	0.544	0.843	0.407	1.745	0.650
		Q3	**0.589**	**0.352**	**0.986**	**0.049**	0.809	0.443	1.478	0.493	0.991	0.489	2.006	0.980
		Q4	0.765	0.439	1.333	0.348	1.292	0.670	2.489	0.448	1.584	0.728	3.448	0.261
	Non-Osteoporosis vs. Osteoporosis	Log2-PPN	1.282	0.961	1.711	0.096	**1.673**	**1.215**	**2.303**	**0.003**	**1.663**	**1.190**	**2.324**	**0.007**
		Q1	Reference				Reference				Reference			
		Q2	1.664	0.868	3.189	0.131	1.794	0.873	3.690	0.118	**2.506**	**1.311**	**4.790**	**0.012**
		Q3	1.049	0.540	2.034	0.889	1.353	0.621	2.951	0.450	1.592	0.739	3.430	0.250
		Q4	1.502	0.793	2.846	0.217	**2.421**	**1.198**	**4.893**	**0.017**	**2.421**	**1.234**	**4.746**	**0.019**
PC	Normal BMD vs. Low BMD	Log2-PC	0.820	0.470	1.429	0.486	1.371	0.705	2.666	0.357	1.668	0.807	3.446	0.181
		Q1	Reference				Reference				Reference			
		Q2	1.127	0.643	1.975	0.677	1.304	0.688	2.471	0.420	1.329	0.739	2.392	0.354
		Q3	0.772	0.430	1.385	0.390	1.276	0.660	2.467	0.471	1.562	0.734	3.327	0.262
		Q4	0.939	0.522	1.690	0.834	1.425	0.696	2.919	0.338	1.664	0.792	3.497	0.194
	Non-Osteoporosis vs. Osteoporosis	Log2-PC	1.194	0.641	2.225	0.579	1.749	0.930	3.290	0.089	1.855	0.998	3.449	0.064
		Q1	Reference				Reference				Reference			
		Q2	1.362	0.762	2.436	0.302	1.549	0.817	2.939	0.186	1.805	0.932	3.498	0.096
		Q3	1.077	0.593	1.959	0.808	1.749	0.890	3.440	0.111	1.974	0.983	3.967	0.071
		Q4	1.070	0.541	2.116	0.847	1.453	0.702	3.008	0.319	1.480	0.746	2.935	0.276
NC	Normal BMD vs. Low BMD	Log2-NC	0.927	0.604	1.423	0.729	1.218	0.715	2.075	0.471	1.310	0.738	2.326	0.368
		Q1	Reference				Reference				Reference			
		Q2	1.012	0.528	1.941	0.971	1.044	0.506	2.155	0.908	1.033	0.484	2.206	0.934
		Q3	0.910	0.466	1.777	0.784	0.966	0.461	2.024	0.927	0.905	0.419	1.951	0.801
		Q4	0.938	0.492	1.790	0.847	1.470	0.662	3.266	0.348	1.623	0.682	3.863	0.288
	Non-Osteoporosis vs. Osteoporosis	Log2-NC	**1.443**	**1.018**	**2.044**	**0.043**	**1.960**	**1.253**	**3.065**	**0.005**	**1.854**	**1.196**	**2.874**	**0.012**
		Q1	Reference				Reference				Reference			
		Q2	1.436	0.740	2.788	0.289	1.578	0.757	3.288	0.229	1.457	0.685	3.100	0.341
		Q3	**2.031**	**1.023**	**4.033**	**0.047**	2.257	1.019	5.003	0.050	**2.120**	**1.126**	**3.991**	**0.031**
		Q4	1.481	0.819	2.677	0.199	**2.198**	**1.147**	**4.210**	**0.021**	1.868	0.955	3.654	0.084
LC	Normal BMD vs. Low BMD	Log2-LC	**0.585**	**0.349**	**0.981**	**0.047**	0.793	0.468	1.342	0.391	0.708	0.449	1.116	0.152
		Q1	Reference				Reference				Reference			
		Q2	0.838	0.424	1.657	0.614	0.886	0.417	1.882	0.754	0.873	0.412	1.850	0.727
		Q3	0.678	0.356	1.292	0.242	0.909	0.440	1.876	0.797	0.896	0.457	1.755	0.752
		Q4	0.606	0.299	1.227	0.169	0.863	0.418	1.781	0.692	0.765	0.414	1.415	0.404
	Non-Osteoporosis vs. Osteoporosis	Log2-LC	1.000	0.646	1.549	0.998	1.430	0.842	2.428	0.191	1.165	0.682	1.988	0.582
		Q1	Reference				Reference				Reference			
		Q2	1.231	0.739	2.050	0.428	1.297	0.742	2.268	0.365	0.957	0.567	1.616	0.871
		Q3	**1.636**	**1.011**	**2.646**	**0.050**	**2.327**	**1.321**	**4.100**	**0.005**	**2.013**	**1.079**	**3.754**	**0.040**
		Q4	1.206	0.605	2.406	0.596	1.841	0.873	3.879	0.115	1.229	0.623	2.426	0.559

SII: Q1 (68.67-289.81), Q2 (290.00-419.38), Q3 (419.76-570.94), Q4 (571.17-5313.00); PLR: Q1 (42.65-93.87), Q2 (94.07-115.77), Q3 (115.79-144.29), Q4 (144.38-690.00); NLR: Q1 (0.333-1.278), Q2 (1.281-1.706), Q3 (1.708-2.241), Q4 (2.250-19.250); PPN: Q1 (151.20-638.60), Q2 (639.60-856.80), Q3 (857.50-1173.00), Q4 (1177.40-7352.40); PC: Q1 (54.00-208.00), Q2 (209.00-241.00), Q3 (242.00-285.00), Q4 (286.00-1000.00); NC: Q1 (0.90-2.70), Q2 (2.80-3.50), Q3 (3.60-4.50), Q4 (4.60-13.20); LC: Q1 (0.40-1.60), Q2 (1.70-2.00), Q3 (2.10-2.50), Q4 (2.60-5.60).

Bold fonts indicate P value < 0.05.

Income level, ALT, AST, blood calcium, serum creatinine, and serum 25(OH)D were categorized into four groups according to the quartiles (Q1-Q4) of distribution.

* Model 1: Unadjusted model.

‡ Model 2: Age (50-64; 65 and over), race (non-Hispanic white; Mexican American; other Hispanic; non-Hispanic black; other races), and BMI (normal; overweight; obese) were adjusted.

¶ Model 3: Age (50-64; 65 and over), race (non-Hispanic white; Mexican American; other Hispanic; non-Hispanic black; other races), education level (under high school; high school or equivalent; above high school), income level (Q1-Q4), BMI (normal; overweight; obese), smoke status (current smokers; quit smoking; never smoke), alcohol consumption (≥ once monthly; < once monthly), diabetes (yes; no; borderline), physical activity level (NMVPA; LMVPA; MMVPA; HMVPA), family history of osteoporosis (yes; no), milk product consumption (never; rarely; sometimes; often; varied), ALT (Q1-Q4); AST (Q1-Q4), blood calcium (Q1-Q4), serum creatinine (Q1-Q4), and serum 25(OH)D (Q1-Q4) were adjusted.

25(OH)D, 25-hydroxyvitamin D; ALT, alanine transaminase; AST, aspartate transaminase; BMD, bone mineral density; BMI, body mass index; CI, confidence interval; FN, femoral neck; HMVPA, high moderate-to-vigorous physical activity; LC, lymphocyte count; LMVPA, low moderate-to-vigorous physical activity; LS, lumbar spine; MMVPA, medium moderate-to-vigorous physical activity; NC, neutrophil count; NLR, neutrophil-to-lymphocyte ratio; NMVPA, no moderate-to-vigorous physical activity; OR, odd ratio; PC, platelet count; PLR, platelet-to-lymphocyte ratio; PPN, the product of platelet count and neutrophil count; SII, systemic immune-inflammation index; TF, total femur.

### Subgroup analysis

The results of the subgroup analysis for the association of BMD with SII and other inflammatory markers among postmenopausal women are listed in **Supplementary Table S3**. The results demonstrated that the negative association between SII and BMD was mainly among women aged ≥ 65 years, women with normal BMI (BMI <25 kg/m2), non-Hispanic white women, or women of other ethnicities (race/ethnicity other than non-Hispanic white, non-Hispanic black, or Mexican American). Moreover, the associations between BMD and other inflammatory markers, especially PPN and NC, were pronounced among postmenopausal women aged ≥65 years or those with normal BMI. In addition, the results of the subgroup analysis for the association of low BMD/osteoporosis with SII and other inflammatory markers among postmenopausal women are listed in **Supplementary Table S4**. The results showed that the association between increased SII and the increased risk of low BMD/osteoporosis was mainly among women aged ≥ 65 years, women with normal BMI (BMI <25 kg/m2), or women of other ethnicities. The subgroup analysis results for other inflammatory markers are displayed in **Supplementary Table S3** and **Supplementary Table S4**.

## Discussion

This study found that SII was negatively associated with total femur BMD and femoral neck BMD, and postmenopausal women in a higher SII quarter group (second quartile and third quartile) showed low lumbar spine BMD than the lowest SII quarter group when SII was converted from a continuous variable to a categorical variable. Moreover, increased SII was associated with an increased risk of low BMD and osteoporosis. In addition, this study observed that other inflammatory markers, especially NLR and PPN, were negatively associated with BMD and positively associated with the risk of osteoporosis among postmenopausal women aged ≥50 years. Finally, the subgroup analysis showed that the associations between BMD and inflammatory markers were pronounced in postmenopausal women aged ≥65 years or those with normal BMI (<25 kg/m^2^).

Several previous studies investigated the relationship between BMD and inflammatory indicators, such as PLR and NLR. Du et al. found that NLR was negatively associated with FN-BMD among Chinese postmenopausal women, but no significant association between NLR and FN-BMD was observed after covariates were adjusted (21). Lee et al. observed that NLR was negatively associated with LS-BMD but not FN-BMD among Korean postmenopausal women, but no significant association between PLR and BMD was observed (29). Moreover, Huang et al. found that increased NLR level was associated with an increased risk of osteoporosis among Chinese postmenopausal women without diabetes (15). Liu et al. demonstrated that increased NLR was associated with an increased risk of osteopenia among Chinese postmenopausal women (30). In addition, a limited number of studies investigated the relationship between SII and bone metabolism. Du et al. found an inverse association between FN-BMD and SII among 413 Chinese postmenopausal women (21). Fang et al. observed that a high SII level (≥ 834.89) was a risk factor for osteoporosis among 238 Chinese postmenopausal women (31). In contrast to the previous study, this study had some advantages. First, previous studies mainly investigated the association of BMD with SII or other inflammatory indicators among Asian populations. However, our study population differed from these studies, and our results provided new evidence on the association of BMD with SII or other inflammatory indicators among the general US population. Second, for a more comprehensive assessment of the association between SII and BMD, this study simultaneously assessed the association of BMD with SII and other inflammatory markers derived from PC, NC, and LC, which was one aspect that differed from other previous studies. Third, previous studies only assessed the association of SII with BMD or osteoporosis/osteopenia risk. However, this study comprehensively analyzed the association of SII with BMD at different sites and the risk of low BMD/osteoporosis in the same population. Finally, this study performed the subgroup analysis to investigate the potential impact of other factors on the association between SII and BMD, which was an essential difference between previous studies and our study.

In the present study, we simultaneously assessed the association of BMD with SII and other inflammatory markers among postmenopausal women aged ≥ 50 years. First, we found that PC, NC, and LC showed limited associations with BMD. Specifically, we only observed that PC was negatively associated with LS-BMD, NC was negatively associated with FN-BMD, and no significant association was observed between LC and BMD at any skeletal sites after adjusting covariates. Moreover, only NC but not PC or LC showed a positive association with the risk of osteoporosis after adjusting covariates. Second, for the inflammatory markers derived by two indicators among PC, NC, and LC (PLR, NLR, or PPN), we observed that increased levels of NLR and PPN (but not PLR) were associated with reduced BMD and the increased risk of osteoporosis. Third, SII, which was derived from PC, NC, and LC, showed not only a significant association with BMD but also the risk of low BMD and osteoporosis. Therefore, SII might be a better inflammatory marker in predicting the risk of osteopenia and osteoporosis among postmenopausal women aged ≥ 50 years from the viewpoint of clinical practice. However, additional large-scale prospective studies are needed to further investigate the role of SII in osteoporosis because of the limited number of related studies and the inherent limitations in the present study.

Interestingly, we also observed a novel marker (PPN) that has not been reported in previous studies on the relationship between inflammatory markers and bone metabolism to our knowledge. When the inflammatory markers were considered a continuous variable, the results suggested that PPN was negatively associated with BMD at any skeletal sites (TF-BMD, FN-BMD, and LS-BMD). In contrast, this study found that SII showed no association with LS-BMD when SII was considered a continuous variable. Moreover, in the subgroup analysis stratified by BMI, we observed that PPN still showed a negative association with LS-BMD among postmenopausal women with normal BMI when PPN was considered a continuous variable. Therefore, PPN might be a useful inflammatory marker to suggest decreased BMD, especially LS-BMD. However, more investigations are required to test the predictive value of PPN for osteoporosis.

The present study observed the main finding that an increased SII was associated with an increased risk of low BMD/osteoporosis among postmenopausal women. It is essential to emphasize the causality between an increased SII and reduced BMD could not be established because of the cross-sectional design of this study. On the one hand, increased SII, which might suggest an elevated inflammatory status or weak immune response, contributed to decreased bone mass. On the other hand, other factors, such as the decline in endogenous estrogen production following menopause, might result in reduced bone mass and changes in inflammatory status and immune response. Although the specific mechanism of the association between increased SII and elevated risk of low BMD/osteoporosis remains unclear, there are several possible explanations that might involve the interaction between the immune and bone systems (9, 11, 32). Neutrophils have been demonstrated to be an essential part of the innate immune system. Previous studies have observed increased neutrophil infiltration in ovariectomized (OVX) mice (33, 34). Moreover, estrogen can affect the functional and physiological activities of neutrophils *in vitro* (9, 35, 36). In addition, neutrophils can contribute to decreased bone mass by expressing mediators that promote bone resorption, such as interleukin 6 (IL-6) and Receptor Activator for Nuclear Factor-κ B (RANKL) (9). Lymphocytes play a key role in adaptive immune response. Several previous studies have demonstrated that lymphocytes also have dual functional roles in bone metabolism because they can regulate the balance between bone formation and bone resorption (9, 11). Moreover, accumulating evidence has shown that lymphocyte number and function are increasing in both postmenopausal females and OVX animals (9, 11, 12, 37). Moreover, lymphocytes, including T and B lymphocytes, have been demonstrated to stimulate osteoclastogenesis through the upregulation of inflammatory factors during postmenopausal osteoporosis (9, 11, 38). The function of platelets in bone metabolism during postmenopausal osteoporosis remains to be elucidated. Previous studies have demonstrated the dual functional roles of platelets. On the one hand, Inflammatory stimulation could stimulate platelet activation, and activated platelets could enhance osteoclastogenesis through activating osteoclastogenic signaling pathways (39, 40). On the other hand, platelets might also have a positive effect on bone remodeling (41). In addition, a study by Ma et al. demonstrated that the circulating platelet count was positively associated with hip and lumbar spine BMD among females (42). Therefore, the association between increased SII and an elevated risk of osteopenia/osteoporosis in postmenopausal women might be explained by the function of neutrophils, lymphocytes, and platelets.

This study observed differences in the association between SII and BMD at different skeletal sites, which suggested that SII was associated with femoral BMD but not lumbar spine BMD. Although the detailed mechanism is uncertain, there are some possible explanations. First, we speculated that the skeletal site difference might be due to the impact of age or BMI. We compared the BMD at different skeletal sites among different age or BMI groups. For age (**Supplementary Table S5**), we found that TF-BMD and FN-BMD were significantly lower among older women than younger women (P-value <0.0001), but no significant differences in LS-BMD between older women and younger women (P-value >0.05). In the subgroup analysis, we only observed that SII was negatively associated with FN-BMD among women aged ≥ 65 after adjusting covariates. For BMI (**Supplementary Table S5**), all BMD at any skeletal site was significantly higher among women with higher BMI than those with lower BMI (P-value <0.001). In the subgroup analysis, we found that SII was negatively associated with TF-BMD and FN-BMD (but not LS-BMD) among women with normal BMI. Therefore, the impact of age and BMI might be a potential reason for the skeletal site difference. Second, we noted that no significant association between LS-BMD and SII was observed when SII was considered a continuous variable. However, the sensitivity analysis showed that postmenopausal women in a higher SII quarter group (second quartile and third quartile) showed low LS-BMD than the lowest SII quarter group. Moreover, we observed an inverted U-shaped relationship between SII and LS-BMD. Therefore, the results of the present study might not be illustrated that there was no association between SII and LS-BMD. Third, there were significant differences in bone structures between the femur and lumbar spine (43, 44). Moreover, there were significant differences in the levels of genes and cells in different skeletal sites (45–47), suggesting that there might be differences in response to the inflammatory stimulus at different skeletal sites. Fourth, inflammatory-related diseases, such as low back pain, were common among older members of the general population (48, 49). We considered that women with inflammatory-related diseases tended to have a high level of inflammatory indicators. However, there might be no differences in LS-BMD between women with inflammatory-related diseases and those without inflammatory-related diseases. For example, Briggs et al. found no differences in total spine aBMD between women with chronic low back pain and those without chronic low back pain (50). Snider et al. observed that individuals with chronic low back pain showed significantly higher LS-BMD than those without chronic low back pain (51). However, given the differences in study design and population, the evidence from previous studies was not sufficient to support our hypothesis, and additional studies are needed in the future to answer this question.

In the present study, we observed that the associations of BMD with SII or some inflammatory markers, such as NLR, were more pronounced in women aged ≥65 years. This study analyzed the inflammatory marker levels in different age groups (**Supplementary Table S6**) and observed that the level of NLR was significantly higher among women aged ≥65 years than those aged 50 to 64. Moreover, age was an independent factor significantly contributing to BMD (52). Therefore, we considered that older women might have a higher level of SII than younger women, which might be one reason for such age difference. However, we also noted that not all inflammatory marker were higher among women aged ≥65 years than those aged 50 to 64, such as PPN. Therefore, further research is required to fully explain this age difference.

The results of the subgroup analysis showed a race difference in the association of SII with BMD or the risk of osteoporosis. First, we considered that the differences in the levels of inflammatory markers and the prevalence of osteoporosis among different race groups might be a potential underlying cause. On the one hand, we noted that the levels of inflammatory markers except for LC seemed higher among non-Hispanic white women than in other races (**Supplementary Table S6**). On the other hand, previous studies have demonstrated the differences in the prevalence of osteoporosis among different races in the US. For example, Looker et al. found that the age-adjusted prevalence of osteoporosis at FN and LS in the US was highest in non-Hispanic Asians, intermediate in non-Hispanic whites and Hispanics, and lowest in non-Hispanic blacks (53). Wright et al. observed the difference in the prevalence of osteoporosis and low bone mass at either FN or LS among women of different ethnicities in the US (non-Hispanic white women: 15.8%; non-Hispanic black women: 7.7%; and Mexican American women: 20.4%) (54). Moreover, we also observed the difference in the prevalence of osteoporosis and osteopenia among women of different ethnicities in the present study (**Supplementary Table S7**). Third, the risk factors for osteoporosis, such as BMI, might differ according to ethnicity and race (55, 56). In the present study, we compared the BMI among women of different ethnicities (**Supplementary Table S8**) and found that BMI was significantly higher among non-Hispanic black or Mexican American women than non-Hispanic white women, while BMI was significantly lower among women of other ethnicities (race/ethnicity other than non-Hispanic white, non-Hispanic black, or Mexican American) compared with non-Hispanic white women. However, further studies are needed owing to the relatively small sample size of the present study.

Subgroup analysis also showed that the association of SII with BMD or the risk of low BMD/osteoporosis was mainly among postmenopausal women with normal BMI but not in overweight or obese subjects. On the one hand, elevated inflammation levels have been demonstrated to be associated with overweight and obese BMI (57). Moreover, we analyzed the levels of inflammatory markers among different BMI groups and found that the levels of SII, PPN, NC, and LC were higher among women with higher BMI than those with lower BMI (**Supplementary Table S6**). In contrast, low BMI was considered an essential risk factor for osteoporosis (1, 6), and the prevalence of postmenopausal women with osteopenia/osteoporosis in this study differed between the BMI groups (**Supplementary Table S7** **).** These hypotheses might explain the differences in the association between increased SII and increased risk of low BMD among the different BMI groups. However, further validation is needed in other studies with larger sample sizes because of the small sample size of the present study.

The main findings of this study could provide valuable suggestions for clinical practice and future research. First, although the causality between SII and BMD could not be assessed because of the cross-sectional study design, the results of the present study suggest that postmenopausal females with high levels of SII or other inflammatory markers should be aware of the potential risk of osteopenia and osteoporosis. Second, as a conventional test, a complete blood count can be conducted in primary health institutions or large hospitals. Therefore, the SII might be considered a novel useful index to initially assess the risk of osteopenia or osteoporosis among postmenopausal women. Third, this study provided a relatively comprehensive view of the relationship between BMD and the inflammatory markers derived from PC, NC, and LC. Fourth, this study found a novel index (PPN) and observed that PPN showed a negative association with BMD at all sites (TF-BMD, FN-BMD, and LS-BMD) when PPN was considered a continuous variable, which is a characteristic that distinguished PPN from SII or other inflammatory markers. Fifth, the subgroup analysis of the present study showed that the associations between BMD and inflammatory markers were pronounced among women aged ≥ 65 years or women with normal BMI, which suggested that some potential factors, such as age and BMI, might modify the association between BMD and inflammatory marker. Therefore, the specific population, such as postmenopausal women aged ≥ 65 years and postmenopausal with normal BMI, should be aware of the potential risk of osteopenia and osteoporosis. Moreover, future studies are warranted to include consideration of age and BMI differences. Finally, considering the inherent limitations of this study, it is worth investigating the potential value of SII in monitoring drug efficacy of anti-osteoporotic agents or fracture risk assessment in further studies.

This study has some limitations. First, causality between SII and BMD could not be established because of the cross-sectional design of this study. Second, although this study performed a weighted analysis, the sample size is relatively small. Therefore, further prospective studies with larger sample sizes are required. Third, some information on covariates was collected based on self-reported questionnaires, which might not accurately reflect the actual situation and introduce recall bias. Finally, some confounding variables (such as C-reactive protein and sex hormone levels) were not included finally because they were not available in the NHANES database.

## Conclusion

SII may be a valuable and convenient inflammatory marker that could be applied to predict the risk of low BMD or osteoporosis among postmenopausal women aged ≥50. Moreover, postmenopausal women with a high level of SII or other inflammatory markers, such as NLR and PPN, should be aware of the potential risk of osteoporosis. However, given the inherent limitations of the present study, additional large-scale studies are required to investigate the role of SII in osteoporosis further.

## Data availability statement

Publicly available datasets were analyzed in this study. This data can be found here: https://www.cdc.gov/nchs/nhanes/.

## Ethics statement

The studies involving human participants were reviewed and approved by The ethics review board of the National Center for Health Statistics. The patients/participants provided their written informed consent to participate in this study.

## Author contributions

YT and BP have contributed equally to this work. YT: Conceptualization, Methodology, Software, Formal analysis, Data Curation, Writing - Original Draft, Writing - Review & Editing, Funding acquisition; BP: Methodology, Formal analysis, Validation, Investigation, Writing - Original Draft, Writing - Review & Editing; JL: Software, Data Curation, Visualization; ZL: Validation, Writing - Review & Editing; YX: Conceptualization, Writing - Review & Editing, Supervision, Funding acquisition; BG: Conceptualization, Methodology, Writing - Review & Editing, Supervision, Funding acquisition. All authors contributed to the article and approved the submitted version.

## Funding

This work was supported by the National Natural Science Foundation of China (81874017; 81960403; 82060405), Cuiying Scientific and Technological Innovation Program of Lanzhou University Second Hospital (CY2017-ZD02; CY2021-MS-A07), and Innovation Star Project for Excellent Graduate Students of the Education Department of Gansu Province (2021CXZX-143).

## Acknowledgments

We would like to thank Editage (www.editage.cn) for English language editing.

## Conflict of interest

The authors declare that the research was conducted in the absence of any commercial or financial relationships that could be construed as a potential conflict of interest.

## Publisher’s note

All claims expressed in this article are solely those of the authors and do not necessarily represent those of their affiliated organizations, or those of the publisher, the editors and the reviewers. Any product that may be evaluated in this article, or claim that may be made by its manufacturer, is not guaranteed or endorsed by the publisher.
