# Supplementary material for: Systemic immune-inflammation index and bone mineral density in postmenopausal women: A cross-sectional study of the national health and nutrition examination survey (NHANES) 2007-2018
Source: Front Immunol. 2022 Sep 8;13:975400. doi: 10.3389/fimmu.2022.975400 (PMC9493473; doi:10.3389/fimmu.2022.975400)
Supplement: Supplementary file 1 [file DataSheet_1.docx]

Supplementary Material

**Supplementary Table S1** Definition of osteopenia and osteoporosis

**Supplementary Table S2** Detailed information on covariates

**Supplementary Table S3** Subgroup analysis for the association of BMD with SII and other inflammatory markers among postmenopausal women

**Supplementary Table S4** Subgroup analysis for the association of low BMD/osteoporosis with SII and other inflammatory markers among postmenopausal women

**Supplementary Table S5** BMD at different skeletal sites among different age or BMI groups

**Supplementary Table S6** SII and other inflammatory markers in different subgroups

**Supplementary Table S7** Prevalence of osteoporosis and osteopenia in different subgroups

**Supplementary Table S8** BMI among different race groups

. **Supplementary Table S1** Definition of osteopenia and osteoporosis

| **Skeletal Site** | **Mean** | **SD** | **Mean-2.5SD** | **Mean-SD** | **Osteoporosis** | **Osteopenia** | **Normal BMD** |
| --- | --- | --- | --- | --- | --- | --- | --- |
| Total Femur (g/cm^2^) | 0.94 | 0.122 | 0.635 | 0.818 | ≤0.635 | 0.635 < and < 0.818 | ≥0.818 |
| Femoral Neck (g/cm^2^) | 0.86 | 0.12 | 0.56 | 0.74 | ≤0.560 | 0.560 < and < 0.740 | ≥0.740 |
| Lumbar Spine (g/cm^2^) | 1.064 | 0.106 | 0.799 | 0.958 | ≤0.799 | 0.799 < and < 0.958 | ≥0.958 |

BMD, bone mineral density, SE, standard deviation.

**Supplementary Table S2** Detailed information on covariates

| **Covariate** | **Range (if continues variable)** | **Grouping for adjustment** |
| --- | --- | --- |
| Age | 50-80 years ("80" means ≥ 80 years of age) | 50-64 years |
|  |  | 65-80+ years |
| Race | NA | Mexican American |
|  |  | Other Hispanic |
|  |  | Non-Hispanic white |
|  |  | Non-Hispanic black |
|  |  | Other races |
| Education level | NA | Under high school |
|  |  | High school or equivalent |
|  |  | Above high school |
| Income level | PIR: 0.00-5.00 ("5.00" means ≥ 5.00) | Q1 (PIR: 0.00-1.10) |
|  |  | Q2 (PIR: 1.11-2.02) |
|  |  | Q3 (PIR: 2.03-3.84) |
|  |  | Q4 (PIR: 3.85-5.00) |
| BMI | 13.18-53.89 kg/m2 | Normal (BMI < 25kg/m2) |
|  |  | Overweight (25 ≤ BMI < 30kg/m2) |
|  |  | Obese (BMI ≥ 30kg/m2) |
| Smoking status | NA | Current smokers |
|  |  | Quit smoking |
|  |  | Never smoke |
| Alcohol consumption | NA | Yes (≥ once monthly) |
|  |  | No (< once monthly) |
| Diabetes | NA | Yes (diagnosed by doctors) |
|  |  | No |
|  |  | Borderline |
| Physical activity level | 0-34800 MET-mins/week | NMVPA (0 MET-mins/week) |
|  |  | LMVPA (1-599 MET-mins/week) |
|  |  | MMVPA (600-1199 MET-mins/week) |
|  |  | HMVPA (≥1200 MET-mins/week) |
| Family history of osteoporosis | NA | Yes (patients with osteoporosis diagnosed by doctors) |
|  |  | No |
| Milk product consumption | NA | Never |
|  |  | Rarely (less than once a week) |
|  |  | Sometimes (once a week or more, but less than once a day) |
|  |  | Often (once a day or more) |
|  |  | Varied |
| ALT | 5.00-225.00 U/L | Q1 (5.00-14.00 U/L) |
|  |  | Q2 (15.00-18.00 U/L) |
|  |  | Q3 (19.00-24.00 U/L) |
|  |  | Q4 (25.00-225.00 U/L) |
| AST | 11.00-112.00 U/L | Q1 (11.00-18.00 U/L) |
|  |  | Q2 (19.00-21.00 U/L) |
|  |  | Q3 (22.00-25.00 U/L) |
|  |  | Q4 (26.00-112.00 U/L) |
| Blood calcium | 8.50-11.30 mg/dL | Q1 (8.50-9.10 mg/dL) |
|  |  | Q2 (9.20-9.40 mg/dL) |
|  |  | Q3 (9.50-9.60 mg/dL) |
|  |  | Q4 (9.70-11.30 mg/dL) |
| Serum creatinine | 0.40-8.32 mg/dL | Q1 (0.40-0.66 mg/dL) |
|  |  | Q2 (0.67-0.75 mg/dL) |
|  |  | Q3 (0.76-0.87 mg/dL) |
|  |  | Q4 (0.88-8.32 mg/dL) |
| Serum 25(OH)D | 13.80-219.00 nmol/L | Q1 (13.80-47.40 nmol/L) |
|  |  | Q2 (47.50-67.40 nmol/L) |
|  |  | Q3 (67.50-85.70 nmol/L) |
|  |  | Q4 (85.90-219.00 nmol/L) |

Income level, ALT, AST, blood calcium, serum creatinine, and serum 25(OH)D were divided into four groups according to the quartiles of distribution.

ALT, alanine transaminase; AST, aspartate transaminase; BMI, body mass index; HMVPA, high moderate-to-vigorous physical activity; LMVPA, low moderate-to-vigorous physical activity; MMVPA, medium moderate-to-vigorous physical activity; NMVPA, no moderate-to-vigorous physical activity; PIR, family income-to-poverty ratio; 25(OH)D, 25-hydroxyvitamin D.

**Supplementary Table S3** Subgroup analysis for the association of BMD with SII and other inflammatory markers among postmenopausal women

| **Index** | **Outcome** | **Subgroup** | | **Model 1 *** | | | | **Model 2 ǂ** | | | | **Model 3 ¶** | | | |
| --- | --- | --- | --- | --- | --- | --- | --- | --- | --- | --- | --- | --- | --- | --- | --- |
|  |  |  |  | **β** | **95%CI low** | **95%CI upp** | ***p*** | **β** | **95%CI low** | **95%CI upp** | ***p*** | **β** | **95%CI low** | **95%CI upp** | ***p*** |
| SII | TF-BMD | Age | 50-64 years old | -0.004 | -0.032 | 0.023 | 0.753 | -0.019 | -0.043 | 0.005 | 0.123 | -0.017 | -0.036 | 0.002 | 0.100 |
|  |  |  | 65-80+ years old | -0.020 | -0.046 | 0.005 | 0.124 | -0.019 | -0.047 | 0.009 | 0.193 | -0.024 | -0.049 | -0.000 | 0.063 |
|  |  | Race | Non-Hispanic white | -0.002 | -0.030 | 0.026 | 0.874 | -0.023 | -0.050 | 0.004 | 0.106 | **-0.024** | **-0.045** | **-0.002** | **0.047** |
|  |  |  | Non-Hispanic black | -0.007 | -0.029 | 0.015 | 0.544 | -0.004 | -0.024 | 0.017 | 0.742 | -0.004 | -0.028 | 0.019 | 0.724 |
|  |  |  | Mexican American | -0.024 | -0.056 | 0.008 | 0.157 | -0.013 | -0.051 | 0.024 | 0.500 | -0.005 | -0.037 | 0.026 | 0.737 |
|  |  |  | Other races ※ | -0.028 | -0.064 | 0.008 | 0.131 | **-0.034** | **-0.064** | **-0.004** | **0.035** | -0.024 | -0.049 | 0.001 | 0.064 |
|  |  | BMI | Normal | **-0.043** | **-0.064** | **-0.022** | **0.000** | **-0.035** | **-0.058** | **-0.012** | **0.004** | **-0.031** | **-0.049** | **-0.014** | **0.003** |
|  |  |  | Overweight | -0.014 | -0.037 | 0.009 | 0.229 | -0.006 | -0.030 | 0.018 | 0.625 | -0.007 | -0.034 | 0.021 | 0.644 |
|  |  |  | Obese | -0.018 | -0.055 | 0.020 | 0.359 | -0.015 | -0.054 | 0.024 | 0.450 | -0.008 | -0.031 | 0.015 | 0.501 |
|  | FN-BMD | Age | 50-64 years old | -0.009 | -0.033 | 0.015 | 0.463 | -0.017 | -0.038 | 0.004 | 0.123 | -0.016 | -0.034 | 0.002 | 0.095 |
|  |  |  | 65-80+ years old | **-0.023** | **-0.044** | **-0.002** | **0.040** | -0.021 | -0.043 | 0.002 | 0.083 | **-0.024** | **-0.044** | **-0.004** | **0.032** |
|  |  | Race | Non-Hispanic white | -0.005 | -0.027 | 0.018 | 0.689 | -0.020 | -0.042 | 0.002 | 0.084 | -0.019 | -0.038 | -0.000 | 0.066 |
|  |  |  | Non-Hispanic black | -0.012 | -0.029 | 0.004 | 0.158 | -0.008 | -0.024 | 0.007 | 0.303 | -0.004 | -0.024 | 0.015 | 0.671 |
|  |  |  | Mexican American | **-0.029** | **-0.051** | **-0.007** | **0.017** | -0.020 | -0.047 | 0.007 | 0.160 | -0.014 | -0.041 | 0.012 | 0.288 |
|  |  |  | Other races ※ | -0.022 | -0.053 | 0.009 | 0.168 | -0.026 | -0.052 | 0.000 | 0.060 | -0.024 | -0.049 | 0.002 | 0.075 |
|  |  | BMI | Normal | **-0.032** | **-0.054** | **-0.009** | **0.007** | -0.023 | -0.047 | -0.000 | 0.054 | **-0.023** | **-0.043** | **-0.003** | **0.033** |
|  |  |  | Overweight | -0.022 | -0.044 | 0.000 | 0.060 | -0.014 | -0.037 | 0.009 | 0.248 | -0.013 | -0.039 | 0.014 | 0.364 |
|  |  |  | Obese | -0.025 | -0.058 | 0.007 | 0.136 | -0.018 | -0.052 | 0.016 | 0.297 | -0.008 | -0.031 | 0.016 | 0.530 |
|  | LS-BMD | Age | 50-64 years old | 0.003 | -0.027 | 0.032 | 0.863 | -0.012 | -0.040 | 0.017 | 0.428 | -0.013 | -0.041 | 0.015 | 0.368 |
|  |  |  | 65-80+ years old | -0.009 | -0.036 | 0.018 | 0.522 | -0.009 | -0.038 | 0.020 | 0.544 | -0.014 | -0.042 | 0.013 | 0.313 |
|  |  | Race | Non-Hispanic white | 0.003 | -0.034 | 0.039 | 0.883 | -0.014 | -0.051 | 0.023 | 0.464 | -0.019 | -0.052 | 0.015 | 0.295 |
|  |  |  | Non-Hispanic black | -0.004 | -0.025 | 0.018 | 0.738 | -0.001 | -0.023 | 0.021 | 0.922 | 0.003 | -0.019 | 0.025 | 0.795 |
|  |  |  | Mexican American | 0.008 | -0.032 | 0.048 | 0.703 | 0.010 | -0.032 | 0.051 | 0.651 | 0.004 | -0.032 | 0.040 | 0.816 |
|  |  |  | Other races ※ | **-0.029** | **-0.057** | **-0.002** | **0.042** | **-0.030** | **-0.058** | **-0.002** | **0.046** | **-0.029** | **-0.052** | **-0.005** | **0.020** |
|  |  | BMI | Normal | -0.030 | -0.068 | 0.008 | 0.130 | -0.025 | -0.064 | 0.014 | 0.214 | -0.024 | -0.051 | 0.003 | 0.097 |
|  |  |  | Overweight | -0.011 | -0.043 | 0.022 | 0.523 | -0.010 | -0.043 | 0.022 | 0.532 | -0.012 | -0.043 | 0.020 | 0.476 |
|  |  |  | Obese | 0.004 | -0.040 | 0.047 | 0.873 | -0.001 | -0.045 | 0.044 | 0.979 | 0.005 | -0.029 | 0.039 | 0.786 |
| PLR | TF-BMD | Age | 50-64 years old | -0.017 | -0.054 | 0.020 | 0.366 | -0.007 | -0.038 | 0.024 | 0.668 | -0.010 | -0.036 | 0.015 | 0.436 |
|  |  |  | 65-80+ years old | -0.036 | -0.107 | 0.034 | 0.313 | -0.024 | -0.078 | 0.031 | 0.394 | -0.030 | -0.063 | 0.003 | 0.096 |
|  |  | Race | Non-Hispanic white | -0.015 | -0.058 | 0.027 | 0.485 | -0.012 | -0.049 | 0.025 | 0.537 | -0.020 | -0.047 | 0.008 | 0.184 |
|  |  |  | Non-Hispanic black | -0.005 | -0.041 | 0.032 | 0.801 | 0.008 | -0.024 | 0.039 | 0.631 | 0.010 | -0.026 | 0.046 | 0.593 |
|  |  |  | Mexican American | **-0.070** | **-0.096** | **-0.045** | **0.000** | **-0.043** | **-0.080** | **-0.007** | **0.031** | -0.039 | -0.080 | 0.001 | 0.058 |
|  |  |  | Other races ※ | **-0.041** | **-0.074** | **-0.008** | **0.019** | -0.030 | -0.065 | 0.004 | 0.097 | -0.007 | -0.039 | 0.025 | 0.666 |
|  |  | BMI | Normal | -0.021 | -0.047 | 0.005 | 0.115 | -0.015 | -0.043 | 0.013 | 0.295 | -0.019 | -0.043 | 0.004 | 0.116 |
|  |  |  | Overweight | 0.010 | -0.014 | 0.034 | 0.405 | 0.012 | -0.013 | 0.037 | 0.340 | 0.013 | -0.015 | 0.042 | 0.375 |
|  |  |  | Obese | -0.041 | -0.103 | 0.020 | 0.194 | -0.042 | -0.110 | 0.025 | 0.227 | **-0.045** | **-0.079** | **-0.012** | **0.016** |
|  | FN-BMD | Age | 50-64 years old | -0.012 | -0.046 | 0.021 | 0.471 | -0.001 | -0.028 | 0.026 | 0.939 | -0.004 | -0.027 | 0.020 | 0.751 |
|  |  |  | 65-80+ years old | -0.029 | -0.075 | 0.016 | 0.210 | -0.020 | -0.055 | 0.014 | 0.254 | -0.024 | -0.047 | -0.002 | 0.050 |
|  |  | Race | Non-Hispanic white | -0.008 | -0.042 | 0.025 | 0.622 | -0.005 | -0.035 | 0.025 | 0.741 | -0.012 | -0.038 | 0.014 | 0.382 |
|  |  |  | Non-Hispanic black | 0.003 | -0.027 | 0.034 | 0.826 | 0.014 | -0.014 | 0.042 | 0.323 | 0.027 | -0.010 | 0.064 | 0.189 |
|  |  |  | Mexican American | **-0.062** | **-0.090** | **-0.034** | **0.000** | **-0.039** | **-0.075** | **-0.003** | **0.045** | -0.029 | -0.065 | 0.006 | 0.111 |
|  |  |  | Other races ※ | **-0.036** | **-0.066** | **-0.007** | **0.022** | **-0.029** | **-0.055** | **-0.003** | **0.036** | -0.017 | -0.047 | 0.014 | 0.285 |
|  |  | BMI | Normal | -0.010 | -0.036 | 0.017 | 0.480 | -0.002 | -0.031 | 0.026 | 0.867 | -0.010 | -0.035 | 0.015 | 0.433 |
|  |  |  | Overweight | 0.004 | -0.019 | 0.028 | 0.725 | 0.006 | -0.019 | 0.030 | 0.657 | 0.005 | -0.022 | 0.033 | 0.714 |
|  |  |  | Obese | -0.032 | -0.081 | 0.017 | 0.212 | -0.029 | -0.082 | 0.025 | 0.297 | -0.031 | -0.067 | 0.005 | 0.112 |
|  | LS-BMD | Age | 50-64 years old | 0.002 | -0.033 | 0.036 | 0.922 | 0.005 | -0.028 | 0.038 | 0.777 | -0.002 | -0.034 | 0.030 | 0.902 |
|  |  |  | 65-80+ years old | -0.009 | -0.079 | 0.060 | 0.793 | -0.004 | -0.061 | 0.052 | 0.878 | 0.000 | -0.040 | 0.041 | 0.990 |
|  |  | Race | Non-Hispanic white | -0.004 | -0.049 | 0.042 | 0.877 | 0.000 | -0.043 | 0.044 | 0.987 | -0.007 | -0.043 | 0.029 | 0.710 |
|  |  |  | Non-Hispanic black | -0.003 | -0.043 | 0.038 | 0.895 | 0.005 | -0.035 | 0.046 | 0.796 | 0.015 | -0.027 | 0.058 | 0.501 |
|  |  |  | Mexican American | -0.027 | -0.074 | 0.021 | 0.281 | -0.007 | -0.053 | 0.038 | 0.752 | -0.017 | -0.061 | 0.028 | 0.459 |
|  |  |  | Other races ※ | -0.002 | -0.065 | 0.060 | 0.940 | 0.002 | -0.055 | 0.059 | 0.942 | -0.006 | -0.040 | 0.028 | 0.739 |
|  |  | BMI | Normal | 0.008 | -0.044 | 0.060 | 0.768 | 0.011 | -0.042 | 0.064 | 0.689 | 0.006 | -0.025 | 0.037 | 0.694 |
|  |  |  | Overweight | 0.024 | -0.019 | 0.067 | 0.275 | 0.021 | -0.023 | 0.066 | 0.357 | 0.013 | -0.026 | 0.053 | 0.516 |
|  |  |  | Obese | -0.020 | -0.088 | 0.047 | 0.554 | -0.032 | -0.099 | 0.034 | 0.342 | -0.037 | -0.087 | 0.013 | 0.165 |
| NLR | TF-BMD | Age | 50-64 years old | -0.011 | -0.039 | 0.018 | 0.460 | -0.015 | -0.039 | 0.009 | 0.223 | -0.013 | -0.034 | 0.007 | 0.218 |
|  |  |  | 65-80+ years old | -0.013 | -0.045 | 0.019 | 0.429 | -0.014 | -0.046 | 0.018 | 0.383 | -0.024 | -0.048 | 0.000 | 0.067 |
|  |  | Race | Non-Hispanic white | 0.001 | -0.030 | 0.031 | 0.967 | -0.012 | -0.040 | 0.016 | 0.388 | -0.014 | -0.035 | 0.007 | 0.202 |
|  |  |  | Non-Hispanic black | **-0.031** | **-0.055** | **-0.007** | **0.015** | -0.018 | -0.040 | 0.004 | 0.122 | -0.021 | -0.046 | 0.003 | 0.129 |
|  |  |  | Mexican American | -0.027 | -0.061 | 0.008 | 0.147 | -0.018 | -0.060 | 0.024 | 0.406 | -0.003 | -0.039 | 0.034 | 0.887 |
|  |  |  | Other races ※ | -0.023 | -0.059 | 0.013 | 0.217 | -0.031 | -0.063 | 0.002 | 0.073 | -0.020 | -0.047 | 0.007 | 0.146 |
|  |  | BMI | Normal | **-0.041** | **-0.069** | **-0.013** | **0.006** | **-0.032** | **-0.061** | **-0.003** | **0.036** | -0.023 | -0.046 | 0.001 | 0.074 |
|  |  |  | Overweight | -0.019 | -0.050 | 0.012 | 0.237 | -0.001 | -0.031 | 0.030 | 0.971 | 0.004 | -0.025 | 0.034 | 0.775 |
|  |  |  | Obese | -0.020 | -0.055 | 0.014 | 0.247 | -0.012 | -0.051 | 0.026 | 0.532 | -0.004 | -0.027 | 0.019 | 0.750 |
|  | FN-BMD | Age | 50-64 years old | -0.018 | -0.043 | 0.007 | 0.161 | -0.016 | -0.038 | 0.007 | 0.180 | -0.013 | -0.033 | 0.007 | 0.208 |
|  |  |  | 65-80+ years old | -0.021 | -0.048 | 0.005 | 0.119 | -0.020 | -0.046 | 0.005 | 0.123 | **-0.028** | **-0.047** | **-0.009** | **0.009** |
|  |  | Race | Non-Hispanic white | -0.004 | -0.029 | 0.021 | 0.751 | -0.013 | -0.037 | 0.011 | 0.286 | -0.011 | -0.031 | 0.008 | 0.276 |
|  |  |  | Non-Hispanic black | **-0.038** | **-0.057** | **-0.019** | **0.000** | **-0.026** | **-0.043** | **-0.009** | **0.005** | **-0.027** | **-0.045** | **-0.009** | **0.018** |
|  |  |  | Mexican American | **-0.039** | **-0.070** | **-0.008** | **0.023** | -0.033 | -0.067 | 0.001 | 0.072 | -0.022 | -0.055 | 0.011 | 0.192 |
|  |  |  | Other races ※ | -0.018 | -0.050 | 0.015 | 0.302 | -0.022 | -0.049 | 0.005 | 0.125 | -0.021 | -0.047 | 0.005 | 0.109 |
|  |  | BMI | Normal | **-0.035** | **-0.066** | **-0.004** | **0.031** | -0.023 | -0.055 | 0.008 | 0.152 | -0.016 | -0.042 | 0.010 | 0.235 |
|  |  |  | Overweight | -0.027 | -0.056 | 0.003 | 0.088 | -0.009 | -0.039 | 0.021 | 0.565 | -0.001 | -0.031 | 0.028 | 0.930 |
|  |  |  | Obese | **-0.032** | **-0.061** | **-0.004** | **0.032** | -0.019 | -0.052 | 0.013 | 0.243 | -0.007 | -0.028 | 0.013 | 0.484 |
|  | LS-BMD | Age | 50-64 years old | 0.003 | -0.032 | 0.037 | 0.877 | -0.002 | -0.036 | 0.032 | 0.910 | -0.003 | -0.034 | 0.029 | 0.867 |
|  |  |  | 65-80+ years old | 0.013 | -0.024 | 0.049 | 0.491 | 0.009 | -0.029 | 0.047 | 0.648 | -0.003 | -0.036 | 0.030 | 0.865 |
|  |  | Race | Non-Hispanic white | 0.018 | -0.027 | 0.063 | 0.441 | 0.006 | -0.038 | 0.050 | 0.777 | 0.001 | -0.033 | 0.036 | 0.949 |
|  |  |  | Non-Hispanic black | -0.010 | -0.037 | 0.017 | 0.467 | -0.000 | -0.026 | 0.025 | 0.997 | 0.002 | -0.022 | 0.025 | 0.901 |
|  |  |  | Mexican American | -0.003 | -0.052 | 0.046 | 0.902 | -0.007 | -0.052 | 0.039 | 0.774 | -0.007 | -0.056 | 0.042 | 0.778 |
|  |  |  | Other races ※ | -0.031 | -0.072 | 0.010 | 0.153 | -0.031 | -0.073 | 0.012 | 0.170 | **-0.039** | **-0.069** | **-0.008** | **0.016** |
|  |  | BMI | Normal | -0.021 | -0.066 | 0.025 | 0.375 | -0.015 | -0.062 | 0.032 | 0.539 | -0.006 | -0.039 | 0.026 | 0.705 |
|  |  |  | Overweight | -0.006 | -0.052 | 0.041 | 0.814 | 0.000 | -0.045 | 0.046 | 0.985 | 0.008 | -0.033 | 0.050 | 0.692 |
|  |  |  | Obese | 0.011 | -0.033 | 0.054 | 0.633 | 0.012 | -0.035 | 0.058 | 0.626 | 0.011 | -0.021 | 0.044 | 0.492 |
| PPN | TF-BMD | Age | 50-64 years old | 0.008 | -0.017 | 0.034 | 0.528 | -0.022 | -0.044 | 0.001 | 0.062 | -0.017 | -0.036 | 0.001 | 0.080 |
|  |  |  | 65-80+ years old | -0.011 | -0.056 | 0.034 | 0.640 | -0.014 | -0.054 | 0.027 | 0.510 | -0.019 | -0.047 | 0.010 | 0.214 |
|  |  | Race | Non-Hispanic white | 0.004 | -0.024 | 0.032 | 0.758 | **-0.029** | **-0.058** | **-0.001** | **0.049** | -0.027 | -0.052 | -0.001 | 0.058 |
|  |  |  | Non-Hispanic black | 0.012 | -0.012 | 0.035 | 0.332 | 0.003 | -0.018 | 0.025 | 0.755 | 0.004 | -0.021 | 0.029 | 0.778 |
|  |  |  | Mexican American | 0.008 | -0.031 | 0.046 | 0.689 | 0.009 | -0.029 | 0.046 | 0.647 | 0.009 | -0.016 | 0.034 | 0.492 |
|  |  |  | Other races ※ | -0.015 | -0.053 | 0.023 | 0.448 | -0.026 | -0.057 | 0.005 | 0.116 | **-0.024** | **-0.047** | **-0.002** | **0.034** |
|  |  | BMI | Normal | **-0.048** | **-0.069** | **-0.026** | **0.000** | **-0.042** | **-0.066** | **-0.018** | **0.001** | **-0.037** | **-0.054** | **-0.020** | **0.000** |
|  |  |  | Overweight | -0.021 | -0.046 | 0.004 | 0.111 | -0.019 | -0.045 | 0.008 | 0.173 | -0.025 | -0.053 | 0.004 | 0.103 |
|  |  |  | Obese | 0.003 | -0.033 | 0.039 | 0.864 | 0.002 | -0.037 | 0.041 | 0.907 | 0.009 | -0.015 | 0.034 | 0.460 |
|  | FN-BMD | Age | 50-64 years old | 0.003 | -0.020 | 0.025 | 0.821 | -0.020 | -0.041 | 0.000 | 0.053 | **-0.020** | **-0.037** | **-0.003** | **0.036** |
|  |  |  | 65-80+ years old | -0.014 | -0.048 | 0.020 | 0.419 | -0.015 | -0.047 | 0.017 | 0.356 | -0.018 | -0.043 | 0.008 | 0.198 |
|  |  | Race | Non-Hispanic white | -0.001 | -0.023 | 0.021 | 0.948 | **-0.028** | **-0.051** | **-0.005** | **0.023** | **-0.025** | **-0.047** | **-0.003** | **0.045** |
|  |  |  | Non-Hispanic black | 0.004 | -0.014 | 0.021 | 0.702 | -0.003 | -0.019 | 0.013 | 0.744 | 0.002 | -0.022 | 0.025 | 0.901 |
|  |  |  | Mexican American | 0.003 | -0.024 | 0.030 | 0.813 | 0.004 | -0.022 | 0.030 | 0.753 | 0.001 | -0.020 | 0.023 | 0.895 |
|  |  |  | Other races ※ | -0.011 | -0.045 | 0.023 | 0.542 | -0.018 | -0.047 | 0.012 | 0.248 | -0.019 | -0.043 | 0.005 | 0.115 |
|  |  | BMI | Normal | **-0.034** | **-0.054** | **-0.015** | **0.001** | **-0.030** | **-0.051** | **-0.008** | **0.008** | **-0.030** | **-0.046** | **-0.014** | **0.002** |
|  |  |  | Overweight | **-0.026** | **-0.047** | **-0.004** | **0.024** | -0.023 | -0.046 | 0.000 | 0.058 | **-0.028** | **-0.053** | **-0.002** | **0.047** |
|  |  |  | Obese | -0.007 | -0.039 | 0.026 | 0.691 | -0.005 | -0.040 | 0.030 | 0.779 | 0.006 | -0.019 | 0.032 | 0.631 |
|  | LS-BMD | Age | 50-64 years old | 0.002 | -0.026 | 0.030 | 0.888 | -0.024 | -0.051 | 0.003 | 0.088 | -0.023 | -0.048 | 0.002 | 0.080 |
|  |  |  | 65-80+ years old | -0.022 | -0.067 | 0.022 | 0.331 | -0.022 | -0.062 | 0.018 | 0.279 | -0.030 | -0.064 | 0.004 | 0.096 |
|  |  | Race | Non-Hispanic white | -0.006 | -0.040 | 0.028 | 0.738 | -0.034 | -0.069 | -0.000 | 0.053 | **-0.038** | **-0.072** | **-0.004** | **0.048** |
|  |  |  | Non-Hispanic black | 0.001 | -0.021 | 0.024 | 0.905 | -0.004 | -0.026 | 0.018 | 0.714 | -0.001 | -0.026 | 0.023 | 0.911 |
|  |  |  | Mexican American | 0.030 | -0.005 | 0.065 | 0.109 | 0.025 | -0.010 | 0.061 | 0.175 | 0.020 | -0.009 | 0.048 | 0.180 |
|  |  |  | Other races ※ | -0.029 | -0.058 | -0.001 | 0.053 | **-0.033** | **-0.062** | **-0.004** | **0.035** | **-0.023** | **-0.045** | **-0.001** | **0.046** |
|  |  | BMI | Normal | **-0.051** | **-0.083** | **-0.018** | **0.003** | **-0.046** | **-0.079** | **-0.013** | **0.009** | **-0.048** | **-0.075** | **-0.020** | **0.003** |
|  |  |  | Overweight | -0.031 | -0.067 | 0.005 | 0.100 | -0.033 | -0.069 | 0.004 | 0.086 | **-0.038** | **-0.070** | **-0.005** | **0.034** |
|  |  |  | Obese | 0.008 | -0.036 | 0.052 | 0.711 | 0.006 | -0.039 | 0.051 | 0.801 | 0.018 | -0.013 | 0.050 | 0.273 |
| PC | TF-BMD | Age | 50-64 years old | 0.015 | -0.045 | 0.074 | 0.630 | -0.026 | -0.075 | 0.024 | 0.313 | -0.022 | -0.059 | 0.015 | 0.261 |
|  |  |  | 65-80+ years old | -0.044 | -0.108 | 0.021 | 0.191 | -0.033 | -0.102 | 0.036 | 0.359 | -0.032 | -0.085 | 0.021 | 0.254 |
|  |  | Race | Non-Hispanic white | -0.009 | -0.069 | 0.051 | 0.764 | -0.044 | -0.099 | 0.011 | 0.122 | -0.041 | -0.085 | 0.004 | 0.095 |
|  |  |  | Non-Hispanic black | **0.084** | **0.034** | **0.134** | **0.002** | **0.050** | **0.005** | **0.096** | **0.035** | 0.056 | 0.006 | 0.106 | 0.061 |
|  |  |  | Mexican American | -0.013 | -0.097 | 0.070 | 0.756 | 0.001 | -0.067 | 0.069 | 0.971 | -0.012 | -0.055 | 0.032 | 0.600 |
|  |  |  | Other races ※ | -0.042 | -0.115 | 0.031 | 0.268 | -0.044 | -0.097 | 0.009 | 0.111 | -0.031 | -0.074 | 0.012 | 0.160 |
|  |  | BMI | Normal | -0.041 | -0.084 | 0.003 | 0.070 | -0.039 | -0.084 | 0.007 | 0.103 | **-0.043** | **-0.071** | **-0.014** | **0.009** |
|  |  |  | Overweight | -0.004 | -0.070 | 0.063 | 0.910 | -0.020 | -0.086 | 0.046 | 0.561 | -0.037 | -0.093 | 0.019 | 0.211 |
|  |  |  | Obese | 0.002 | -0.080 | 0.084 | 0.960 | -0.019 | -0.104 | 0.066 | 0.665 | -0.021 | -0.074 | 0.033 | 0.456 |
|  | FN-BMD | Age | 50-64 years old | 0.019 | -0.033 | 0.071 | 0.473 | -0.017 | -0.062 | 0.028 | 0.465 | -0.020 | -0.058 | 0.018 | 0.313 |
|  |  |  | 65-80+ years old | -0.030 | -0.085 | 0.025 | 0.294 | -0.024 | -0.082 | 0.035 | 0.432 | -0.019 | -0.064 | 0.027 | 0.430 |
|  |  | Race | Non-Hispanic white | -0.005 | -0.058 | 0.048 | 0.857 | -0.033 | -0.081 | 0.015 | 0.185 | -0.033 | -0.078 | 0.011 | 0.162 |
|  |  |  | Non-Hispanic black | **0.084** | **0.040** | **0.128** | **0.001** | **0.056** | **0.017** | **0.095** | **0.007** | **0.077** | **0.031** | **0.124** | **0.011** |
|  |  |  | Mexican American | 0.003 | -0.058 | 0.063 | 0.935 | 0.015 | -0.031 | 0.061 | 0.529 | 0.007 | -0.038 | 0.052 | 0.768 |
|  |  |  | Other races ※ | -0.035 | -0.096 | 0.027 | 0.274 | -0.036 | -0.083 | 0.010 | 0.136 | -0.027 | -0.073 | 0.019 | 0.246 |
|  |  | BMI | Normal | -0.018 | -0.060 | 0.023 | 0.390 | -0.020 | -0.062 | 0.022 | 0.356 | -0.032 | -0.063 | -0.002 | 0.052 |
|  |  |  | Overweight | -0.011 | -0.067 | 0.045 | 0.699 | -0.027 | -0.083 | 0.029 | 0.353 | -0.045 | -0.094 | 0.004 | 0.090 |
|  |  |  | Obese | 0.016 | -0.061 | 0.094 | 0.684 | -0.007 | -0.089 | 0.075 | 0.863 | -0.006 | -0.063 | 0.052 | 0.852 |
|  | LS-BMD | Age | 50-64 years old | 0.002 | -0.056 | 0.059 | 0.958 | -0.035 | -0.088 | 0.018 | 0.201 | -0.039 | -0.082 | 0.004 | 0.093 |
|  |  |  | 65-80+ years old | **-0.069** | **-0.132** | **-0.007** | **0.034** | -0.058 | -0.121 | 0.006 | 0.084 | -0.050 | -0.114 | 0.014 | 0.146 |
|  |  | Race | Non-Hispanic white | -0.036 | -0.094 | 0.022 | 0.228 | **-0.063** | **-0.118** | **-0.007** | **0.033** | **-0.064** | **-0.111** | **-0.017** | **0.019** |
|  |  |  | Non-Hispanic black | 0.020 | -0.035 | 0.075 | 0.483 | -0.005 | -0.060 | 0.049 | 0.848 | 0.008 | -0.050 | 0.067 | 0.787 |
|  |  |  | Mexican American | 0.037 | -0.026 | 0.100 | 0.265 | 0.050 | -0.009 | 0.108 | 0.112 | 0.032 | -0.015 | 0.079 | 0.190 |
|  |  |  | Other races ※ | -0.030 | -0.085 | 0.026 | 0.301 | -0.031 | -0.077 | 0.015 | 0.201 | -0.008 | -0.048 | 0.033 | 0.712 |
|  |  | BMI | Normal | -0.046 | -0.102 | 0.009 | 0.105 | -0.044 | -0.099 | 0.012 | 0.130 | **-0.056** | **-0.100** | **-0.013** | **0.021** |
|  |  |  | Overweight | -0.023 | -0.100 | 0.054 | 0.558 | -0.037 | -0.109 | 0.034 | 0.314 | **-0.068** | **-0.127** | **-0.008** | **0.038** |
|  |  |  | Obese | -0.026 | -0.101 | 0.049 | 0.497 | -0.044 | -0.123 | 0.035 | 0.277 | -0.021 | -0.089 | 0.048 | 0.558 |
| NC | TF-BMD | Age | 50-64 years old | 0.008 | -0.023 | 0.039 | 0.610 | -0.027 | -0.055 | 0.001 | 0.062 | -0.020 | -0.046 | 0.005 | 0.137 |
|  |  |  | 65-80+ years old | 0.002 | -0.062 | 0.065 | 0.954 | -0.009 | -0.061 | 0.043 | 0.733 | -0.018 | -0.054 | 0.018 | 0.341 |
|  |  | Race | Non-Hispanic white | 0.013 | -0.022 | 0.049 | 0.460 | -0.029 | -0.065 | 0.007 | 0.118 | -0.024 | -0.057 | 0.009 | 0.171 |
|  |  |  | Non-Hispanic black | -0.008 | -0.034 | 0.018 | 0.533 | -0.010 | -0.036 | 0.015 | 0.435 | -0.014 | -0.042 | 0.015 | 0.383 |
|  |  |  | Mexican American | 0.022 | -0.017 | 0.061 | 0.278 | 0.017 | -0.026 | 0.060 | 0.454 | 0.024 | -0.010 | 0.057 | 0.166 |
|  |  |  | Other races ※ | -0.010 | -0.059 | 0.038 | 0.677 | -0.032 | -0.078 | 0.015 | 0.188 | **-0.037** | **-0.069** | **-0.006** | **0.022** |
|  |  | BMI | Normal | **-0.067** | **-0.099** | **-0.036** | **0.000** | **-0.057** | **-0.088** | **-0.025** | **0.001** | **-0.045** | **-0.071** | **-0.019** | **0.003** |
|  |  |  | Overweight | **-0.039** | **-0.066** | **-0.011** | **0.008** | -0.027 | -0.058 | 0.004 | 0.096 | -0.029 | -0.064 | 0.006 | 0.117 |
|  |  |  | Obese | 0.004 | -0.034 | 0.042 | 0.835 | 0.011 | -0.033 | 0.055 | 0.624 | 0.024 | -0.008 | 0.056 | 0.157 |
|  | FN-BMD | Age | 50-64 years old | -0.004 | -0.031 | 0.023 | 0.750 | **-0.029** | **-0.054** | **-0.003** | **0.031** | **-0.026** | **-0.048** | **-0.003** | **0.039** |
|  |  |  | 65-80+ years old | -0.011 | -0.056 | 0.034 | 0.627 | -0.017 | -0.055 | 0.021 | 0.394 | -0.023 | -0.054 | 0.009 | 0.172 |
|  |  | Race | Non-Hispanic white | 0.001 | -0.025 | 0.028 | 0.919 | **-0.032** | **-0.061** | **-0.004** | **0.033** | -0.025 | -0.051 | 0.002 | 0.089 |
|  |  |  | Non-Hispanic black | **-0.022** | **-0.041** | **-0.002** | **0.038** | **-0.022** | **-0.041** | **-0.003** | **0.029** | -0.025 | -0.051 | 0.002 | 0.108 |
|  |  |  | Mexican American | 0.005 | -0.027 | 0.037 | 0.763 | 0.000 | -0.033 | 0.034 | 0.980 | -0.001 | -0.031 | 0.029 | 0.964 |
|  |  |  | Other races ※ | -0.005 | -0.050 | 0.040 | 0.835 | -0.019 | -0.064 | 0.027 | 0.429 | -0.028 | -0.061 | 0.005 | 0.096 |
|  |  | BMI | Normal | **-0.055** | **-0.087** | **-0.024** | **0.001** | **-0.045** | **-0.077** | **-0.014** | **0.007** | **-0.038** | **-0.064** | **-0.011** | **0.011** |
|  |  |  | Overweight | **-0.045** | **-0.068** | **-0.021** | **0.001** | **-0.032** | **-0.059** | **-0.005** | **0.024** | -0.031 | -0.064 | 0.001 | 0.072 |
|  |  |  | Obese | -0.016 | -0.050 | 0.018 | 0.350 | -0.005 | -0.045 | 0.034 | 0.790 | 0.013 | -0.019 | 0.045 | 0.448 |
|  | LS-BMD | Age | 50-64 years old | 0.003 | -0.031 | 0.037 | 0.867 | -0.026 | -0.059 | 0.007 | 0.133 | -0.022 | -0.056 | 0.011 | 0.210 |
|  |  |  | 65-80+ years old | -0.007 | -0.068 | 0.054 | 0.832 | -0.013 | -0.066 | 0.040 | 0.645 | -0.030 | -0.072 | 0.012 | 0.178 |
|  |  | Race | Non-Hispanic white | 0.009 | -0.035 | 0.053 | 0.681 | -0.028 | -0.072 | 0.017 | 0.229 | -0.029 | -0.073 | 0.014 | 0.203 |
|  |  |  | Non-Hispanic black | -0.004 | -0.034 | 0.026 | 0.783 | -0.005 | -0.035 | 0.025 | 0.745 | -0.005 | -0.036 | 0.026 | 0.752 |
|  |  |  | Mexican American | 0.039 | -0.011 | 0.088 | 0.138 | 0.024 | -0.023 | 0.071 | 0.327 | 0.022 | -0.026 | 0.070 | 0.380 |
|  |  |  | Other races ※ | -0.045 | -0.089 | -0.001 | 0.053 | **-0.055** | **-0.104** | **-0.005** | **0.037** | **-0.048** | **-0.084** | **-0.011** | **0.012** |
|  |  | BMI | Normal | **-0.069** | **-0.115** | **-0.023** | **0.005** | **-0.063** | **-0.109** | **-0.016** | **0.010** | **-0.057** | **-0.095** | **-0.019** | **0.009** |
|  |  |  | Overweight | **-0.049** | **-0.092** | **-0.005** | **0.034** | -0.046 | -0.095 | 0.002 | 0.068 | -0.039 | -0.083 | 0.004 | 0.092 |
|  |  |  | Obese | 0.023 | -0.027 | 0.072 | 0.371 | 0.026 | -0.027 | 0.080 | 0.337 | 0.039 | -0.000 | 0.077 | 0.066 |
| LC | TF-BMD | Age | 50-64 years old | 0.026 | -0.006 | 0.057 | 0.114 | -0.007 | -0.033 | 0.019 | 0.608 | -0.001 | -0.022 | 0.021 | 0.938 |
|  |  |  | 65-80+ years old | 0.021 | -0.072 | 0.114 | 0.660 | 0.011 | -0.062 | 0.085 | 0.766 | 0.020 | -0.015 | 0.056 | 0.270 |
|  |  | Race | Non-Hispanic white | 0.012 | -0.030 | 0.054 | 0.581 | -0.010 | -0.051 | 0.031 | 0.624 | 0.000 | -0.028 | 0.028 | 0.983 |
|  |  |  | Non-Hispanic black | **0.058** | **0.016** | **0.100** | **0.009** | 0.023 | -0.011 | 0.057 | 0.199 | 0.023 | -0.015 | 0.061 | 0.278 |
|  |  |  | Mexican American | **0.064** | **0.029** | **0.100** | **0.002** | 0.044 | -0.003 | 0.092 | 0.083 | 0.033 | -0.008 | 0.074 | 0.114 |
|  |  |  | Other races ※ | 0.016 | -0.022 | 0.055 | 0.412 | 0.004 | -0.035 | 0.043 | 0.845 | -0.012 | -0.040 | 0.016 | 0.409 |
|  |  | BMI | Normal | -0.006 | -0.043 | 0.032 | 0.768 | -0.012 | -0.054 | 0.029 | 0.560 | -0.011 | -0.040 | 0.018 | 0.458 |
|  |  |  | Overweight | -0.013 | -0.043 | 0.017 | 0.402 | -0.022 | -0.052 | 0.007 | 0.146 | **-0.035** | **-0.063** | **-0.007** | **0.025** |
|  |  |  | Obese | 0.043 | -0.012 | 0.097 | 0.128 | 0.035 | -0.032 | 0.102 | 0.315 | **0.035** | **0.008** | **0.063** | **0.020** |
|  | FN-BMD | Age | 50-64 years old | 0.023 | -0.007 | 0.053 | 0.134 | -0.008 | -0.033 | 0.017 | 0.516 | -0.007 | -0.028 | 0.014 | 0.519 |
|  |  |  | 65-80+ years old | 0.020 | -0.043 | 0.082 | 0.539 | 0.012 | -0.038 | 0.062 | 0.641 | 0.022 | -0.005 | 0.048 | 0.126 |
|  |  | Race | Non-Hispanic white | 0.007 | -0.026 | 0.040 | 0.688 | -0.012 | -0.044 | 0.020 | 0.455 | -0.005 | -0.031 | 0.020 | 0.686 |
|  |  |  | Non-Hispanic black | **0.048** | **0.016** | **0.081** | **0.006** | 0.018 | -0.008 | 0.045 | 0.174 | 0.016 | -0.019 | 0.052 | 0.391 |
|  |  |  | Mexican American | **0.065** | **0.024** | **0.105** | **0.005** | 0.048 | 0.003 | 0.093 | 0.052 | 0.035 | -0.004 | 0.074 | 0.086 |
|  |  |  | Other races ※ | 0.016 | -0.017 | 0.049 | 0.345 | 0.007 | -0.022 | 0.036 | 0.629 | -0.001 | -0.027 | 0.025 | 0.931 |
|  |  | BMI | Normal | -0.002 | -0.041 | 0.036 | 0.899 | -0.013 | -0.052 | 0.026 | 0.507 | -0.014 | -0.043 | 0.015 | 0.352 |
|  |  |  | Overweight | -0.010 | -0.039 | 0.020 | 0.521 | -0.018 | -0.048 | 0.012 | 0.237 | **-0.029** | **-0.057** | **-0.002** | **0.048** |
|  |  |  | Obese | 0.040 | -0.002 | 0.081 | 0.065 | 0.026 | -0.024 | 0.077 | 0.314 | 0.028 | 0.000 | 0.056 | 0.063 |
|  | LS-BMD | Age | 50-64 years old | -0.001 | -0.042 | 0.040 | 0.964 | -0.025 | -0.066 | 0.015 | 0.230 | -0.020 | -0.051 | 0.012 | 0.245 |
|  |  |  | 65-80+ years old | -0.026 | -0.123 | 0.071 | 0.603 | -0.026 | -0.106 | 0.053 | 0.520 | -0.034 | -0.091 | 0.023 | 0.261 |
|  |  | Race | Non-Hispanic white | -0.015 | -0.071 | 0.041 | 0.594 | -0.035 | -0.089 | 0.019 | 0.211 | -0.029 | -0.070 | 0.012 | 0.191 |
|  |  |  | Non-Hispanic black | 0.016 | -0.032 | 0.063 | 0.523 | -0.010 | -0.051 | 0.031 | 0.633 | -0.013 | -0.053 | 0.028 | 0.559 |
|  |  |  | Mexican American | **0.048** | **0.004** | **0.091** | **0.043** | 0.036 | -0.007 | 0.079 | 0.122 | 0.038 | -0.011 | 0.087 | 0.128 |
|  |  |  | Other races ※ | -0.015 | -0.076 | 0.047 | 0.649 | -0.020 | -0.078 | 0.038 | 0.506 | 0.001 | -0.032 | 0.033 | 0.965 |
|  |  | BMI | Normal | -0.047 | -0.100 | 0.006 | 0.085 | -0.051 | -0.106 | 0.005 | 0.081 | **-0.055** | **-0.090** | **-0.019** | **0.007** |
|  |  |  | Overweight | -0.037 | -0.095 | 0.022 | 0.227 | -0.040 | -0.097 | 0.018 | 0.180 | -0.051 | -0.104 | 0.003 | 0.078 |
|  |  |  | Obese | 0.009 | -0.062 | 0.080 | 0.812 | 0.013 | -0.061 | 0.086 | 0.735 | 0.027 | -0.013 | 0.066 | 0.203 |

SII, PLR, NLR, PPN, PC, NC, and LC were considered continuous variables (log2-SII, log2-PLR, log2-NLR, log2-PPN, log2-PC, log2-NC, log2-LC).

Bold fonts indicate P value < 0.05.

SII, PLR, NLR, PPN, PC, NC, and LC were log2-transformed in regression analysis.

Income level, ALT, AST, blood calcium, serum creatinine, and serum 25(OH)D were categorized into four groups according to the quartiles (Q1-Q4) of distribution.

※ “Other races” includes race/ethnicity other than non-Hispanic white, non-Hispanic black, or Mexican American.

* Model 1: Unadjusted model.

ǂ Model 2: Age (50-64; 65 and over. Age was not adjusted when stratified by age), race (non-Hispanic white; Mexican American; other Hispanic; non-Hispanic black; other races. Race was not adjusted when stratified by race), and BMI (normal; overweight; obese. BMI was not adjusted when stratified by BMI) were adjusted.

¶ Model 3: Age (50-64; 65 and over. Age was not adjusted when stratified by age), race (non-Hispanic white; Mexican American; other Hispanic; non-Hispanic black; other races. Race was not adjusted when stratified by race), education level (under high school; high school or equivalent; above high school), income level (Q1-Q4), BMI (normal; overweight; obese. BMI was not adjusted when stratified by BMI), smoke status (current smokers; quit smoking; never smoke), alcohol consumption (≥ once monthly; < once monthly), diabetes (yes; no; borderline), physical activity level (NMVPA; LMVPA; MMVPA; HMVPA), family history of osteoporosis (yes; no), milk product consumption (never; rarely; sometimes; often; varied), ALT (Q1-Q4); AST (Q1-Q4), blood calcium (Q1-Q4), serum creatinine (Q1-Q4), and serum 25(OH)D (Q1-Q4) were adjusted.

25(OH)D, 25-hydroxyvitamin D; ALT, alanine transaminase; AST, aspartate transaminase; BMD, bone mineral density; BMI, body mass index; CI, confidence interval; FN, femoral neck; HMVPA, high moderate-to-vigorous physical activity; LC, lymphocyte count; LMVPA, low moderate-to-vigorous physical activity; LS, lumbar spine; MMVPA, medium moderate-to-vigorous physical activity; NC, neutrophil count; NLR, neutrophil-to-lymphocyte ratio; NMVPA, no moderate-to-vigorous physical activity; PC, platelet count; PLR, platelet-to-lymphocyte ratio; PPN, the product of platelet count and neutrophil count; SII, systemic immune-inflammation index; TF, total femur.

**Supplementary Table S4** Subgroup analysis for the association of low BMD/osteoporosis with SII and other inflammatory markers among postmenopausal women

| **Index** | **Outcome** | **Subgroup** | | **Model 1 *** | | | | **Model 2 ǂ** | | | | **Model 3 ¶** | | | |
| --- | --- | --- | --- | --- | --- | --- | --- | --- | --- | --- | --- | --- | --- | --- | --- |
|  |  |  |  | **OR** | **95%CI low** | **95%CI upp** | ***p*** | **OR** | **95%CI low** | **95%CI upp** | ***p*** | **OR** | **95%CI low** | **95%CI upp** | ***p*** |
| SII | Normal BMD  vs.  Low BMD | Age | 50-64 years old | 1.118 | 0.790 | 1.584 | 0.531 | 1.347 | 0.906 | 2.004 | 0.147 | 1.433 | 0.896 | 2.292 | 0.149 |
|  |  |  | 65-80+ years old | 1.324 | 0.803 | 2.181 | 0.276 | 1.406 | 0.802 | 2.463 | 0.240 | **2.136** | **1.256** | **3.634** | **0.012** |
|  |  | Race | Non-Hispanic white | 1.113 | 0.722 | 1.715 | 0.629 | 1.410 | 0.857 | 2.321 | 0.182 | 1.638 | 0.898 | 2.988 | 0.130 |
|  |  |  | Non-Hispanic black | 1.117 | 0.795 | 1.570 | 0.527 | 1.112 | 0.741 | 1.669 | 0.611 | 1.089 | 0.704 | 1.682 | 0.712 |
|  |  |  | Mexican American | 1.242 | 0.817 | 1.886 | 0.322 | 1.281 | 0.763 | 2.150 | 0.361 | 2.191 | 0.799 | 6.008 | 0.131 |
|  |  |  | Other races ※ | 1.806 | 0.870 | 3.750 | 0.122 | 2.042 | 0.896 | 4.650 | 0.099 | 1.158 | 0.350 | 3.830 | 0.810 |
|  |  | BMI | Normal | 1.662 | 0.529 | 5.222 | 0.388 | 1.560 | 0.449 | 5.423 | 0.487 | **4.160** | **1.142** | **15.154** | **0.044** |
|  |  |  | Overweight | 1.614 | 0.909 | 2.864 | 0.108 | 1.645 | 0.926 | 2.924 | 0.096 | 1.917 | 1.009 | 3.640 | 0.061 |
|  |  |  | Obese | 1.212 | 0.750 | 1.959 | 0.436 | 1.143 | 0.703 | 1.859 | 0.593 | 1.101 | 0.638 | 1.899 | 0.734 |
|  | Non-Osteoporosis  vs.  Osteoporosis | Age | 50-64 years old | 1.215 | 0.821 | 1.799 | 0.335 | 1.451 | 0.924 | 2.279 | 0.112 | 1.402 | 0.926 | 2.123 | 0.126 |
|  |  |  | 65-80+ years old | 1.291 | 0.926 | 1.801 | 0.138 | 1.282 | 0.862 | 1.906 | 0.226 | 1.287 | 0.768 | 2.158 | 0.351 |
|  |  | Race | Non-Hispanic white | 1.061 | 0.712 | 1.581 | 0.773 | 1.255 | 0.802 | 1.964 | 0.326 | 1.535 | 0.906 | 2.598 | 0.133 |
|  |  |  | Non-Hispanic black | **1.839** | **1.118** | **3.025** | **0.021** | 1.789 | 0.968 | 3.305 | 0.071 | 16.986 | 0.835 | 345.589 | 0.103 |
|  |  |  | Mexican American | 1.539 | 0.854 | 2.774 | 0.166 | 1.290 | 0.634 | 2.625 | 0.491 | 1.211 | 0.571 | 2.566 | 0.619 |
|  |  |  | Other races ※ | **1.852** | **1.099** | **3.123** | **0.027** | **1.978** | **1.133** | **3.454** | **0.022** | **2.753** | **1.508** | **5.028** | **0.001** |
|  |  | BMI | Normal | **2.080** | **1.259** | **3.436** | **0.006** | **1.938** | **1.077** | **3.487** | **0.032** | **2.675** | **1.398** | **5.120** | **0.008** |
|  |  |  | Overweight | 1.162 | 0.725 | 1.861 | 0.535 | 1.077 | 0.608 | 1.909 | 0.799 | 1.084 | 0.576 | 2.040 | 0.806 |
|  |  |  | Obese | 1.043 | 0.584 | 1.865 | 0.887 | 1.022 | 0.498 | 2.097 | 0.953 | 1.124 | 0.616 | 2.052 | 0.707 |
| PLR | Normal BMD  vs.  Low BMD | Age | 50-64 years old | 1.501 | 0.931 | 2.422 | 0.101 | 1.422 | 0.830 | 2.436 | 0.205 | 1.645 | 0.893 | 3.031 | 0.126 |
|  |  |  | 65-80+ years old | 1.459 | 0.401 | 5.310 | 0.569 | 1.424 | 0.477 | 4.249 | 0.529 | 1.939 | 0.896 | 4.198 | 0.110 |
|  |  | Race | Non-Hispanic white | 1.452 | 0.799 | 2.637 | 0.227 | 1.403 | 0.716 | 2.750 | 0.329 | 1.685 | 0.831 | 3.414 | 0.170 |
|  |  |  | Non-Hispanic black | 1.029 | 0.616 | 1.719 | 0.912 | 0.920 | 0.534 | 1.583 | 0.764 | 0.828 | 0.397 | 1.730 | 0.629 |
|  |  |  | Mexican American | **2.769** | **1.466** | **5.230** | **0.005** | **2.350** | **1.170** | **4.719** | **0.027** | **7.950** | **1.650** | **38.301** | **0.011** |
|  |  |  | Other races ※ | **3.162** | **1.095** | **9.137** | **0.041** | 2.648 | 0.961 | 7.295 | 0.069 | 1.284 | 0.214 | 7.698 | 0.785 |
|  |  | BMI | Normal | 1.996 | 0.638 | 6.243 | 0.240 | 1.892 | 0.598 | 5.986 | 0.283 | **8.079** | **1.290** | **50.598** | **0.038** |
|  |  |  | Overweight | 0.987 | 0.532 | 1.829 | 0.966 | 1.013 | 0.530 | 1.936 | 0.970 | 1.282 | 0.582 | 2.823 | 0.544 |
|  |  |  | Obese | 1.704 | 0.755 | 3.846 | 0.205 | 1.777 | 0.716 | 4.410 | 0.221 | **2.358** | **1.073** | **5.183** | **0.046** |
|  | Non-Osteoporosis  vs.  Osteoporosis | Age | 50-64 years old | 1.000 | 0.539 | 1.853 | 0.999 | 0.887 | 0.434 | 1.812 | 0.744 | 0.932 | 0.486 | 1.789 | 0.834 |
|  |  |  | 65-80+ years old | 1.254 | 0.734 | 2.141 | 0.411 | 1.153 | 0.699 | 1.902 | 0.580 | 1.104 | 0.492 | 2.478 | 0.813 |
|  |  | Race | Non-Hispanic white | 0.957 | 0.552 | 1.659 | 0.875 | 0.882 | 0.475 | 1.636 | 0.692 | 1.298 | 0.713 | 2.362 | 0.408 |
|  |  |  | Non-Hispanic black | 1.624 | 0.746 | 3.536 | 0.229 | 1.243 | 0.531 | 2.911 | 0.619 | 12.983 | 0.899 | 187.600 | 0.097 |
|  |  |  | Mexican American | 2.123 | 0.821 | 5.491 | 0.135 | 1.613 | 0.590 | 4.415 | 0.363 | 1.909 | 0.629 | 5.794 | 0.257 |
|  |  |  | Other races ※ | 1.304 | 0.614 | 2.767 | 0.495 | 0.355 | 0.603 | 3.048 | 0.467 | 1.534 | 0.638 | 3.689 | 0.341 |
|  |  | BMI | Normal | 0.962 | 0.460 | 2.010 | 0.917 | 0.873 | 0.376 | 2.029 | 0.754 | 1.317 | 0.625 | 2.775 | 0.478 |
|  |  |  | Overweight | 1.106 | 0.613 | 1.993 | 0.740 | 0.124 | 0.569 | 2.219 | 0.738 | 1.190 | 0.497 | 2.850 | 0.701 |
|  |  |  | Obese | 0.891 | 0.418 | 1.897 | 0.766 | 0.988 | 0.457 | 2.135 | 0.976 | 1.398 | 0.617 | 3.168 | 0.432 |
| NLR | Normal BMD  vs.  Low BMD | Age | 50-64 years old | 1.276 | 0.823 | 1.979 | 0.281 | 1.311 | 0.805 | 2.136 | 0.282 | 1.288 | 0.760 | 2.182 | 0.359 |
|  |  |  | 65-80+ years old | 1.308 | 0.705 | 2.428 | 0.398 | 1.460 | 0.759 | 2.806 | 0.262 | **2.709** | **1.421** | **5.166** | **0.007** |
|  |  | Race | Non-Hispanic white | 1.050 | 0.626 | 1.761 | 0.855 | 1.175 | 0.666 | 2.071 | 0.581 | 1.286 | 0.682 | 2.426 | 0.450 |
|  |  |  | Non-Hispanic black | 1.528 | 0.995 | 2.348 | 0.060 | 1.423 | 0.855 | 2.368 | 0.182 | 1.486 | 0.927 | 2.384 | 0.139 |
|  |  |  | Mexican American | **1.771** | **1.059** | **2.961** | **0.040** | **2.032** | **1.333** | **3.096** | **0.004** | 2.785 | 0.842 | 9.210 | 0.097 |
|  |  |  | Other races ※ | 2.747 | 0.776 | 9.721 | 0.126 | 3.012 | 0.844 | 10.747 | 0.099 | 4.741 | 0.922 | 24.384 | 0.064 |
|  |  | BMI | Normal | 1.864 | 0.436 | 7.965 | 0.404 | 1.666 | 0.375 | 7.405 | 0.506 | 5.294 | 1.091 | 25.692 | 0.053 |
|  |  |  | Overweight | 2.004 | 0.838 | 4.791 | 0.124 | 1.827 | 0.792 | 4.213 | 0.164 | 1.793 | 0.834 | 3.855 | 0.152 |
|  |  |  | Obese | 1.228 | 0.727 | 2.075 | 0.446 | 1.071 | 0.617 | 1.860 | 0.809 | 1.111 | 0.637 | 1.936 | 0.715 |
|  | Non-Osteoporosis  vs.  Osteoporosis | Age | 50-64 years old | 1.280 | 0.841 | 1.947 | 0.255 | 1.341 | 0.775 | 2.318 | 0.299 | 1.339 | 0.818 | 2.190 | 0.259 |
|  |  |  | 65-80+ years old | 1.084 | 0.731 | 1.607 | 0.689 | 1.092 | 0.697 | 1.711 | 0.703 | 1.144 | 0.697 | 1.877 | 0.600 |
|  |  | Race | Non-Hispanic white | 1.040 | 0.659 | 1.643 | 0.866 | 1.111 | 0.664 | 1.858 | 0.691 | 1.284 | 0.720 | 2.290 | 0.411 |
|  |  |  | Non-Hispanic black | **2.119** | **1.100** | **4.080** | **0.030** | 1.836 | 0.881 | 3.826 | 0.113 | 137.032 | 0.361 | 51951.751 | 0.143 |
|  |  |  | Mexican American | 1.557 | 0.821 | 2.953 | 0.189 | 1.293 | 0.561 | 2.978 | 0.553 | 1.201 | 0.527 | 2.740 | 0.664 |
|  |  |  | Other races ※ | 1.638 | 0.898 | 2.985 | 0.116 | 1.721 | 0.850 | 3.487 | 0.142 | **2.588** | **1.230** | **5.445** | **0.013** |
|  |  | BMI | Normal | 1.795 | 0.968 | 3.331 | 0.069 | 1.622 | 0.820 | 3.211 | 0.171 | 2.034 | 1.011 | 4.092 | 0.061 |
|  |  |  | Overweight | 1.355 | 0.691 | 2.657 | 0.381 | 1.108 | 0.475 | 2.588 | 0.813 | 0.937 | 0.418 | 2.104 | 0.877 |
|  |  |  | Obese | 1.022 | 0.615 | 1.697 | 0.934 | 0.867 | 0.467 | 1.609 | 0.653 | 0.870 | 0.441 | 1.713 | 0.691 |
| PPN | Normal BMD  vs.  Low BMD | Age | 50-64 years old | 0.839 | 0.607 | 1.160 | 0.293 | 1.189 | 0.817 | 1.732 | 0.370 | 1.285 | 0.827 | 1.998 | 0.278 |
|  |  |  | 65-80+ years old | 1.160 | 0.567 | 2.372 | 0.687 | 1.179 | 0.559 | 2.488 | 0.667 | 1.733 | 0.940 | 3.195 | 0.095 |
|  |  | Race | Non-Hispanic white | 0.946 | 0.599 | 1.495 | 0.814 | 1.412 | 0.848 | 2.351 | 0.191 | 1.721 | 0.838 | 3.536 | 0.161 |
|  |  |  | Non-Hispanic black | 0.900 | 0.634 | 1.278 | 0.560 | 0.982 | 0.645 | 1.497 | 0.935 | 0.936 | 0.554 | 1.584 | 0.812 |
|  |  |  | Mexican American | 0.612 | 0.347 | 1.079 | 0.104 | 0.592 | 0.326 | 1.073 | 0.100 | 0.892 | 0.344 | 2.311 | 0.814 |
|  |  |  | Other races ※ | 0.927 | 0.568 | 1.513 | 0.764 | 0.939 | 0.553 | 1.597 | 0.819 | 0.459 | 0.185 | 1.138 | 0.095 |
|  |  | BMI | Normal | 1.224 | 0.469 | 3.197 | 0.682 | 1.149 | 0.366 | 3.601 | 0.813 | 2.086 | 0.636 | 6.843 | 0.240 |
|  |  |  | Overweight | 1.559 | 0.987 | 2.462 | 0.062 | **1.683** | **1.035** | **2.737** | **0.041** | **2.189** | **1.167** | **4.108** | **0.025** |
|  |  |  | Obese | 0.942 | 0.568 | 1.563 | 0.819 | 0.917 | 0.521 | 1.613 | 0.765 | 0.720 | 0.417 | 1.244 | 0.253 |
|  | Non-Osteoporosis  vs.  Osteoporosis | Age | 50-64 years old | 1.205 | 0.839 | 1.730 | 0.317 | **1.799** | **1.261** | **2.568** | **0.002** | **1.672** | **1.147** | **2.437** | **0.015** |
|  |  |  | 65-80+ years old | 1.399 | 0.906 | 2.160 | 0.135 | 1.453 | 0.918 | 2.299 | 0.117 | 1.520 | 0.822 | 2.808 | 0.198 |
|  |  | Race | Non-Hispanic white | 1.121 | 0.751 | 1.674 | 0.578 | **1.632** | **1.056** | **2.522** | **0.032** | 1.699 | 0.951 | 3.037 | 0.095 |
|  |  |  | Non-Hispanic black | 1.665 | 1.005 | 2.759 | 0.054 | **2.126** | **1.206** | **3.751** | **0.013** | 4.899 | 1.191 | 20.146 | 0.059 |
|  |  |  | Mexican American | 1.149 | 0.664 | 1.986 | 0.624 | 1.081 | 0.589 | 1.986 | 0.803 | 0.968 | 0.443 | 2.115 | 0.935 |
|  |  |  | Other races ※ | **1.851** | **1.110** | **3.087** | **0.024** | **2.050** | **1.170** | **3.592** | **0.017** | **2.752** | **1.506** | **5.027** | **0.001** |
|  |  | BMI | Normal | **3.330** | **2.135** | **5.196** | **0.000** | **3.205** | **1.976** | **5.198** | **0.000** | **3.543** | **2.042** | **6.146** | **0.000** |
|  |  |  | Overweight | 1.017 | 0.604 | 1.713 | 0.951 | 1.002 | 0.564 | 1.780 | 0.994 | 1.140 | 0.578 | 2.250 | 0.710 |
|  |  |  | Obese | 1.142 | 0.622 | 2.095 | 0.671 | 1.177 | 0.558 | 2.482 | 0.671 | 1.217 | 0.705 | 2.101 | 0.489 |
| PC | Normal BMD  vs.  Low BMD | Age | 50-64 years old | 0.746 | 0.365 | 1.523 | 0.424 | 1.368 | 0.581 | 3.223 | 0.476 | 1.823 | 0.725 | 4.582 | 0.216 |
|  |  |  | 65-80+ years old | 1.395 | 0.623 | 3.122 | 0.421 | 1.238 | 0.444 | 3.451 | 0.685 | 1.139 | 0.302 | 4.290 | 0.850 |
|  |  | Race | Non-Hispanic white | 1.262 | 0.542 | 2.938 | 0.592 | 2.165 | 0.800 | 5.860 | 0.135 | 3.113 | 0.851 | 11.389 | 0.108 |
|  |  |  | Non-Hispanic black | **0.352** | **0.150** | **0.827** | **0.021** | 0.445 | 0.178 | 1.111 | 0.090 | 0.350 | 0.106 | 1.149 | 0.122 |
|  |  |  | Mexican American | 0.453 | 0.149 | 1.371 | 0.175 | **0.226** | **0.066** | **0.770** | **0.028** | 0.368 | 0.027 | 5.098 | 0.458 |
|  |  |  | Other races ※ | 0.745 | 0.123 | 4.504 | 0.751 | 0.714 | 0.126 | 4.034 | 0.706 | **0.067** | **0.006** | **0.799** | **0.034** |
|  |  | BMI | Normal | 1.130 | 0.252 | 5.059 | 0.874 | 1.143 | 0.227 | 5.754 | 0.872 | 1.721 | 0.276 | 10.722 | 0.568 |
|  |  |  | Overweight | 1.028 | 0.381 | 2.771 | 0.957 | 1.395 | 0.522 | 3.724 | 0.510 | 2.520 | 0.771 | 8.236 | 0.143 |
|  |  |  | Obese | 1.043 | 0.470 | 2.316 | 0.918 | 1.377 | 0.588 | 3.224 | 0.464 | 1.044 | 0.424 | 2.569 | 0.927 |
|  | Non-Osteoporosis  vs.  Osteoporosis | Age | 50-64 years old | 0.997 | 0.427 | 2.328 | 0.994 | 1.599 | 0.716 | 3.570 | 0.257 | 1.451 | 0.647 | 3.253 | 0.377 |
|  |  |  | 65-80+ years old | 2.171 | 0.986 | 4.780 | 0.059 | 2.085 | 0.864 | 5.032 | 0.108 | 2.127 | 0.580 | 7.796 | 0.270 |
|  |  | Race | Non-Hispanic white | 1.101 | 0.464 | 2.610 | 0.828 | 1.599 | 0.703 | 3.637 | 0.268 | 1.974 | 0.811 | 4.802 | 0.156 |
|  |  |  | Non-Hispanic black | 1.238 | 0.362 | 4.231 | 0.735 | 2.028 | 0.685 | 6.008 | 0.209 | 2.260 | 0.280 | 18.231 | 0.466 |
|  |  |  | Mexican American | 1.409 | 0.415 | 4.780 | 0.588 | 1.259 | 0.397 | 3.989 | 0.700 | 1.245 | 0.289 | 5.362 | 0.770 |
|  |  |  | Other races ※ | **2.857** | **1.059** | **7.704** | **0.045** | **3.445** | **1.295** | **9.160** | **0.019** | **3.481** | **1.079** | **11.226** | **0.038** |
|  |  | BMI | Normal | **2.410** | **1.070** | **5.426** | **0.038** | 2.447 | 0.962 | 6.224 | 0.066 | **3.393** | **1.367** | **8.425** | **0.016** |
|  |  |  | Overweight | 0.800 | 0.363 | 1.759 | 0.580 | 1.024 | 0.457 | 2.294 | 0.954 | 1.762 | 0.546 | 5.690 | 0.356 |
|  |  |  | Obese | 1.110 | 0.320 | 3.848 | 0.870 | 1.717 | 0.466 | 6.322 | 0.420 | 2.632 | 0.727 | 9.528 | 0.157 |
| NC | Normal BMD  vs.  Low BMD | Age | 50-64 years old | 0.838 | 0.529 | 1.329 | 0.456 | 1.174 | 0.679 | 2.029 | 0.569 | 1.160 | 0.630 | 2.134 | 0.639 |
|  |  |  | 65-80+ years old | 1.115 | 0.374 | 3.325 | 0.846 | 1.206 | 0.417 | 3.492 | 0.731 | 2.290 | 1.030 | 5.092 | 0.057 |
|  |  | Race | Non-Hispanic white | 0.793 | 0.395 | 1.590 | 0.516 | 1.251 | 0.577 | 2.709 | 0.573 | 1.432 | 0.533 | 3.845 | 0.488 |
|  |  |  | Non-Hispanic black | 1.172 | 0.743 | 1.850 | 0.498 | 1.237 | 0.709 | 2.159 | 0.458 | 1.311 | 0.701 | 2.450 | 0.421 |
|  |  |  | Mexican American | 0.582 | 0.287 | 1.181 | 0.148 | 0.721 | 0.350 | 1.486 | 0.386 | 1.047 | 0.302 | 3.633 | 0.943 |
|  |  |  | Other races ※ | 0.983 | 0.424 | 2.275 | 0.968 | 1.071 | 0.361 | 3.181 | 0.902 | 1.033 | 0.194 | 5.498 | 0.970 |
|  |  | BMI | Normal | 1.363 | 0.332 | 5.589 | 0.669 | 1.179 | 0.246 | 5.641 | 0.838 | 3.345 | 0.559 | 20.009 | 0.201 |
|  |  |  | Overweight | **2.402** | **1.284** | **4.493** | **0.008** | **2.430** | **1.206** | **4.894** | **0.016** | **2.771** | **1.134** | **6.770** | **0.038** |
|  |  |  | Obese | 0.898 | 0.454 | 1.774 | 0.757 | 0.769 | 0.347 | 1.705 | 0.520 | 0.578 | 0.290 | 1.149 | 0.134 |
|  | Non-Osteoporosis  vs.  Osteoporosis | Age | 50-64 years old | 1.405 | 0.893 | 2.211 | 0.147 | **2.339** | **1.323** | **4.135** | **0.005** | **2.246** | **1.345** | **3.751** | **0.006** |
|  |  |  | 65-80+ years old | 1.269 | 0.744 | 2.163 | 0.386 | 1.403 | 0.791 | 2.489 | 0.252 | 1.544 | 0.740 | 3.221 | 0.262 |
|  |  | Race | Non-Hispanic white | 1.175 | 0.736 | 1.875 | 0.503 | **1.868** | **1.049** | **3.325** | **0.039** | 1.695 | 0.869 | 3.308 | 0.144 |
|  |  |  | Non-Hispanic black | **2.204** | **1.173** | **4.141** | **0.018** | **0.958** | **1.325** | **6.604** | **0.012** | **11.285** | **2.422** | **52.571** | **0.015** |
|  |  |  | Mexican American | 1.098 | 0.559 | 2.159 | 0.789 | 1.031 | 0.474 | 2.243 | 0.940 | 0.825 | 0.295 | 2.307 | 0.715 |
|  |  |  | Other races ※ | **2.150** | **1.033** | **4.476** | **0.048** | **2.539** | **1.056** | **6.106** | **0.045** | **4.515** | **1.917** | **10.634** | **0.001** |
|  |  | BMI | Normal | **5.463** | **2.473** | **12.067** | **0.000** | **4.910** | **2.208** | **10.921** | **0.000** | **5.625** | **2.610** | **12.119** | **0.000** |
|  |  |  | Overweight | 1.162 | 0.534 | 2.530 | 0.706 | 0.991 | 0.403 | 2.437 | 0.985 | 0.946 | 0.350 | 2.554 | 0.914 |
|  |  |  | Obese | 1.183 | 0.635 | 2.204 | 0.599 | 1.046 | 0.443 | 2.469 | 0.919 | 0.918 | 0.452 | 1.867 | 0.816 |
| LC | Normal BMD  vs.  Low BMD | Age | 50-64 years old | **0.555** | **0.335** | **0.921** | **0.026** | 0.803 | 0.485 | 1.332 | 0.400 | 0.783 | 0.467 | 1.315 | 0.367 |
|  |  |  | 65-80+ years old | 0.759 | 0.154 | 3.749 | 0.737 | 0.733 | 0.201 | 2.674 | 0.640 | 0.422 | 0.176 | 1.010 | 0.068 |
|  |  | Race | Non-Hispanic white | 0.748 | 0.405 | 1.381 | 0.358 | 0.996 | 0.516 | 1.923 | 0.990 | 0.948 | 0.517 | 1.740 | 0.866 |
|  |  |  | Non-Hispanic black | **0.496** | **0.260** | **0.948** | **0.040** | 0.667 | 0.337 | 1.323 | 0.253 | 0.681 | 0.295 | 1.570 | 0.394 |
|  |  |  | Mexican American | **0.222** | **0.105** | **0.470** | **0.001** | **0.259** | **0.133** | **0.506** | **0.001** | **0.175** | **0.041** | **0.741** | **0.020** |
|  |  |  | Other races ※ | **0.243** | **0.067** | **0.887** | **0.039** | 0.292 | 0.083 | 1.023 | 0.063 | **0.156** | **0.041** | **0.603** | **0.008** |
|  |  | BMI | Normal | 0.457 | 0.115 | 1.821 | 0.272 | 0.476 | 0.116 | 1.944 | 0.306 | 0.149 | 0.016 | 1.358 | 0.108 |
|  |  |  | Overweight | 1.028 | 0.492 | 2.146 | 0.943 | 1.141 | 0.560 | 2.325 | 0.718 | 1.240 | 0.523 | 2.941 | 0.631 |
|  |  |  | Obese | 0.593 | 0.244 | 1.442 | 0.254 | 0.642 | 0.243 | 1.696 | 0.376 | **0.411** | **0.185** | **0.911** | **0.041** |
|  | Non-Osteoporosis  vs.  Osteoporosis | Age | 50-64 years old | 0.999 | 0.568 | 1.757 | 0.996 | 1.529 | 0.741 | 3.156 | 0.256 | 1.341 | 0.666 | 2.700 | 0.420 |
|  |  |  | 65-80+ years old | 1.143 | 0.546 | 2.396 | 0.724 | 1.266 | 0.662 | 2.421 | 0.479 | 1.382 | 0.576 | 3.316 | 0.478 |
|  |  | Race | Non-Hispanic white | 1.106 | 0.603 | 2.030 | 0.746 | 1.550 | 0.767 | 3.134 | 0.228 | 1.102 | 0.481 | 2.524 | 0.822 |
|  |  |  | Non-Hispanic black | 0.646 | 0.259 | 1.612 | 0.355 | 1.176 | 0.479 | 2.886 | 0.726 | 0.013 | 0.000 | 2.180 | 0.135 |
|  |  |  | Mexican American | 0.565 | 0.222 | 1.436 | 0.243 | 0.696 | 0.233 | 2.079 | 0.524 | 0.546 | 0.162 | 1.833 | 0.330 |
|  |  |  | Other races ※ | 1.315 | 0.671 | 2.575 | 0.430 | 1.495 | 0.682 | 3.274 | 0.323 | 0.367 | 0.602 | 3.102 | 0.456 |
|  |  | BMI | Normal | 2.139 | 0.996 | 4.596 | 0.056 | 2.485 | 0.978 | 6.311 | 0.061 | 1.659 | 0.853 | 3.224 | 0.152 |
|  |  |  | Overweight | 0.810 | 0.372 | 1.765 | 0.598 | 0.888 | 0.355 | 2.220 | 0.800 | 1.052 | 0.355 | 3.117 | 0.928 |
|  |  |  | Obese | 1.181 | 0.584 | 2.386 | 0.645 | 1.279 | 0.597 | 2.740 | 0.530 | 1.156 | 0.477 | 2.801 | 0.752 |

SII, PLR, NLR, PPN, PC, NC, and LC were considered continuous variables (log2-SII, log2-PLR, log2-NLR, log2-PPN, log2-PC, log2-NC, log2-LC).

Bold fonts indicate P value < 0.05.

SII, PLR, NLR, PPN, PC, NC, and LC were log2-transformed in regression analysis.

Income level, ALT, AST, blood calcium, serum creatinine, and serum 25(OH)D were categorized into four groups according to the quartiles (Q1-Q4) of distribution.

※ Other races included race/ethnicity other than non-Hispanic white, non-Hispanic black, or Mexican American.

* Model 1: Unadjusted model.

ǂ Model 2: Age (50-64; 65 and over. Age was not adjusted when stratified by age), race (non-Hispanic white; Mexican American; other Hispanic; non-Hispanic black; other races. Race was not adjusted when stratified by race), and BMI (normal; overweight; obese. BMI was not adjusted when stratified by BMI) were adjusted.

¶ Model 3: Age (50-64; 65 and over. Age was not adjusted when stratified by age), race (non-Hispanic white; Mexican American; other Hispanic; non-Hispanic black; other races. Race was not adjusted when stratified by race), education level (under high school; high school or equivalent; above high school), income level (Q1-Q4), BMI (normal; overweight; obese. BMI was not adjusted when stratified by BMI), smoke status (current smokers; quit smoking; never smoke), alcohol consumption (≥ once monthly; < once monthly), diabetes (yes; no; borderline), physical activity level (NMVPA; LMVPA; MMVPA; HMVPA), family history of osteoporosis (yes; no), milk product consumption (never; rarely; sometimes; often; varied), ALT (Q1-Q4); AST (Q1-Q4), blood calcium (Q1-Q4), serum creatinine (Q1-Q4), and serum 25(OH)D (Q1-Q4) were adjusted.

25(OH)D, 25-hydroxyvitamin D; ALT, alanine transaminase; AST, aspartate transaminase; BMD, bone mineral density; BMI, body mass index; CI, confidence interval; FN, femoral neck; HMVPA, high moderate-to-vigorous physical activity; LC, lymphocyte count; LMVPA, low moderate-to-vigorous physical activity; LS, lumbar spine; MMVPA, medium moderate-to-vigorous physical activity; NC, neutrophil count; NLR, neutrophil-to-lymphocyte ratio; NMVPA, no moderate-to-vigorous physical activity; OR, odd ratio; PC, platelet count; PLR, platelet-to-lymphocyte ratio; PPN, the product of platelet count and neutrophil count; SII, systemic immune-inflammation index; TF, total femur.

**Supplementary Table S5** BMD at different skeletal sites among different age or BMI groups

| **Subgroup** | | **TF-BMD** | **FN-BMD** | **LS-BMD** |
| --- | --- | --- | --- | --- |
| Age | 50-64 years old (reference group) | 0.86 (0.01) | 0.72 (0.01) | 0.94 (0.01) |
|  | 65-80+ years old | 0.80 (0.01) ** | 0.66 (0.01) ** | 0.91 (0.01) |
| BMI | Normal (reference group) | 0.77 (0.01) | 0.65 (0.01) | 0.87 (0.01) |
|  | Overweight | 0.83 (0.01) ** | 0.70 (0.01) ** | 0.93 (0.01) ** |
|  | Obese | 0.94 (0.01) ** | 0.78 (0.01) ** | 1.01 (0.01) ** |

** P-value < 0.01 compared with the reference group.

BMD, bone mineral density; BMI, body mass index; FN, femoral neck; LS, lumbar spine; SE, standard error; TF, total femur.

**Supplementary Table S6** SII and other inflammatory markers in different subgroups

| **Subgroup** | | **SII** | **PLR** | **NLR** | **PPN** | **PC** | **NC** | **LC** |
| --- | --- | --- | --- | --- | --- | --- | --- | --- |
|  |  | **Mean (SE)** | **Mean (SE)** | **Mean (SE)** | **Mean (SE)** | **Mean (SE)** | **Mean (SE)** | **P-value** |
| Age | 50-64 years old ǂ | 474.99 (15.07) | 127.01 (3.30) | 1.89 (0.05) | 976.69 (30.08) | 252.75 (4.16) | 3.78 (0.08) | 2.16 (0.05) |
|  | 65-80+ years old | 524.30 (20.43) | 129.44 (4.63) | 2.13 (0.07) ** | 1017.47 (41.31) | 243.73 (5.12) | 4.06 (0.12) | 2.08 (0.08) |
| Race | Non-Hispanic white ǂ | 514.33 (17.37) | 131.81 (3.59) | 2.07 (0.07) | 1007.25 (33.20) | 249.83 (4.74) | 3.96 (0.09) | 2.07 (0.06) |
|  | Non-Hispanic black | 425.38 (28.81) ** | 119.90 (3.91) * | 1.57 (0.10) ** | 962.32 (52.33) | 268.01 (5.88) * | 3.45 (0.12) ** | 2.43 (0.05) ** |
|  | Mexican American | 473.66 (20.14) | 114.67 (3.24) ** | 1.93 (0.08) | 1042.12 (46.75) | 245.64 (4.93) | 4.15 (0.15) | 2.30 (0.08) ** |
|  | Other races ¶ | 422.63 (24.27) ** | 120.61 (4.51) * | 1.74 (0.06) ** | 888.91 (58.83) | 240.32 (6.91) | 3.56 (0.13) * | 2.16 (0.05) |
| BMI | Normal ǂ | 466.24 (20.27) | 133.66 (4.30) | 1.94 (0.08) | 854.43 (38.65) | 240.72 (7.22) | 3.46 (0.10) | 1.92 (0.05) |
|  | Overweight | 464.38 (18.14) | 123.53 (3.78) * | 1.82 (0.07) | 1000.01 (35.29) * | 255.15 (4.93) | 3.83 (0.10) * | 2.29 (0.08) ** |
|  | Obese | 531.23 (23.31) * | 125.75 (4.61) | 2.10 (0.08) | 1103.97 (41.95) ** | 255.29 (4.72) | 4.26 (0.11) ** | 2.21 (0.08) ** |

ǂ Reference group.

* P < 0.05 compared with the reference group.

** P < 0.01 compared with the reference group.

¶ Other races: race/ethnicity other than non-Hispanic white, non-Hispanic black, or Mexican American.

BMD, bone mineral density; BMI, body mass index; FN, femoral neck; LC, lymphocyte count; LS, lumbar spine; NC, neutrophil count; NLR, neutrophil-to-lymphocyte ratio; PC, platelet count; PLR, platelet-to-lymphocyte ratio; PPN, the product of platelet count and neutrophil count; SE, standard error; SII, systemic immune-inflammation index; TF, total femur.

**Supplementary Table S7** Prevalence of osteoporosis and osteopenia in different subgroups

| **Subgroup** |  | **Normal BMD**  **% (SE)** | **Osteopenia**  **% (SE)** | **Osteoporosis**  **% (SE)** |
| --- | --- | --- | --- | --- |
| Age | 50-64 years old | 31.06 (2.19) | 50.52 (2.41) | 18.42 (2.33) |
|  | 65-80+ years old | 20.66 (2.23) | 44.09 (3.39) | 35.25 (3.69) |
| Race | Non-Hispanic white | 28.67 (2.68) | 48.65 (2.87) | 22.68 (2.48) |
|  | Non-Hispanic black | 46.96 (4.62) | 41.76 (4.00) | 11.28 (2.76) |
|  | Mexican American | 23.15 (3.42) | 46.29 (4.12) | 30.56 (4.65) |
|  | Other races ¶ | 13.43 (2.45) | 57.14 (4.73) | 29.43 (4.59) |
| BMI | Normal | 9.92 (2.86) | 53.57 (4.60) | 36.51 (4.17) |
|  | Overweight | 28.99 (3.53) | 48.36 (4.13) | 22.64 (3.42) |
|  | Obese | 45.71 (3.45) | 44.75 (3.71) | 9.54 (1.75) |

¶ Other race: race/ethnicity other than non-Hispanic white, non-Hispanic black, or Mexican American.

BMD, bone mineral density; BMI, body mass index; SE, standard error;.

**Supplementary Table S8** BMI among different race groups

| **Race group** | **BMI, Mean (SE)** |
| --- | --- |
| Non-Hispanic white | 28.10 (0.44) |
| Non-Hispanic black | 30.43 (0.48) ** |
| Mexican American | 29.89 (0.47) ** |
| Other races ¶ | 26.04 (0.42) ** |

** P < 0.01 compared with non-Hispanic white women.

¶ Other race: race/ethnicity other than non-Hispanic white, non-Hispanic black, or Mexican American.

BMI, body mass index; SE, standard error.
